# Integrated Phenotypic, Physiological, Biochemical, and Transcriptomic Analyses Reveal the Molecular Response Mechanisms of *Populus* to Poplar Canker

**DOI:** 10.3390/jof12010003

**Published:** 2025-12-20

**Authors:** Dongchen Shen, Hui Lin, Yaru Gu, Jian Diao, Ling Ma

**Affiliations:** 1 Forest Protection, College of Forestry, Northeast Forestry University, Harbin 150040, China; 2023010054@nefu.edu.cn (D.S.); lh2023110207@nefu.edu.cn (H.L.); coraline_@nefu.edu.cn (Y.G.); 2 College of Wildlife and Protected Area, Northeast Forestry University, Harbin 150040, China

**Keywords:** poplar, *Botryosphaeria dothidea*, physiology, WGCNA, *PP2C* gene family

## Abstract

The growth process of poplar faces severe environmental challenges. Notably, poplar canker, caused by *Botryosphaeria dothidea*, has significantly impaired poplar productivity and ecological functions. However, research on the molecular mechanisms underlying poplar resistance to this disease remains incomplete. This study systematically elucidated the molecular mechanisms of *Populus davidiana* × *P. alba* var. *pyramidalis* (Pdpap) in response to *B. dothidea* stress by integrating phenotypic, physiological, biochemical, and transcriptomic analyses. The results demonstrated that 5 d post-inoculation with *B. dothidea*, the stem wound sites darkened and developed lesions. Following pathogen infection, H_2_O_2_ content and SOD and POD activity initially increased then decreased, while MDA content overall showed a declining trend with prolonged infection time. KEGG enrichment analysis revealed that DEGs were significantly enriched in the MAPK signaling pathway, plant hormone signal transduction, and phenylpropanoid biosynthesis pathways. Gene modules significantly associated with physiological indices were screened using WGCNA. Within these modules, hub genes in the regulatory network were further identified, leading to the selection of *P2C76*. The genome-wide identification of *PtrPP2Cs* classified 124 members into 13 subgroups. Collectively, this study dissects the gene expression regulation and molecular defense mechanisms of poplar under *B. dothidea* infection, providing novel molecular insights for its molecular breeding.

## 1. Introduction

*Botryosphaeria dothidea*, as the type species of the genus *Botryosphaeria*, is a significant phytopathogenic fungus. This species exhibits a global distribution and an exceptionally broad host range. *B. dothidea* typically infects hosts through wounds or natural openings [1,2]. When plant tissues are healthy, *B. dothidea* persists endophytically, but when hosts become physiologically compromised, it transitions to a pathogenic state [3,4]. This pathogen causes diverse stem diseases in woody plants including gumming, cankers, dieback, and trunk rot. Discovered diseases include trunk canker in Chinese hickory (*Carya cathayensis*) [5], poplar canker, apple branch warts [6], and peach gummosis [7]. Furthermore, *B. dothidea* infects the leaves of vines and shrubs, inducing leaf spot on *Euonymus japonicus* [8] and *Euonymus fortunei* [9] while concurrently causing leaf blight on *Parrotia subaequalis* [10] and *Syringa oblata* [11]. Simultaneously, this pathogen triggers fruit rot and ring spot, along with crop diseases including nut rot in *Prunus dulcis* [12], root rot in sugar beet (*Beta vulgaris*) [13], fruit soft rot in *Pyracantha fortuneana* [14], and apple ring rot [15]. Consequently, *B. dothidea* poses a severe potential threat to agricultural and forestry ecosystems. Current research on *B. dothidea*–host interactions primarily focuses on *Malus domestica*-*B. dothidea* [16], *Pyrus bretschneideri*-*B. dothidea* [17], and *C. cathayensis*-*B. dothidea* [18] pathosystems, while the molecular interaction mechanisms between poplar and *B. dothidea* remain poorly characterized.

Poplar (*Populus* spp.) is one of the most important broad-leaved tree species for plantations in temperate regions worldwide [19]. Valued for its rapid growth, wide adaptability, and diverse economic benefits, it holds significant importance in carbon sink forestry [20], ecological restoration [21,22], and economic development [23]. However, poplar canker severely constrains its utilization value. This disease primarily damages the branches and trunks of poplars, affecting trees from saplings to maturity, with young trees being particularly vulnerable. It often leads to complete dieback and is one of the most destructive diseases affecting poplars. The pathogens causing poplar canker are diverse and mainly fall into two categories: fungal cankers and bacterial cankers [24]. Currently identified poplar cankers are predominantly caused by fungi [25]. Among the fungal cankers, *B. dothidea* causes the most severe losses. Early control strategies for poplar canker caused by *B. dothidea* relied primarily on chemical methods [26]. However, the increasing prominence of pesticide resistance and environmental pollution resulting from long-term pesticide use has shifted research focus in recent years towards biological control and resistance breeding. Although biological control offers advantages such as a low risk of inducing resistance, high selectivity, and environmental friendliness, it still faces limitations including the slow onset of action, unstable efficacy, and relatively high costs [27]. In contrast, resistance breeding not only possesses the advantages of biological control but also demonstrates durable resistance, stable effectiveness, and lower costs [28]. Therefore, breeding poplar varieties with enhanced resistance to canker disease is crucial. Elucidating the molecular mechanisms by which *B. dothidea* interferes with poplar defense pathways and regulatory networks will provide a critical theoretical foundation for the precise breeding of disease-resistant varieties.

With the rapid advancement of high-throughput sequencing technologies, RNA-seq has been extensively applied to decipher transcriptomic variations in diverse organisms under specific conditions, enabling an in-depth exploration of molecular mechanisms, regulatory networks, and key candidate genes [29]. In recent years, this technology has played a pivotal role in elucidating molecular mechanisms underlying plant responses to both biotic and abiotic stresses [30]. Researchers conducting transcriptomic analysis on *Zanthoxylum bungeanum* with varying drought tolerance revealed that drought-resistant strains enhance tolerance through coordinated mechanisms, including elevated antioxidant enzyme activity, an intensified pentose phosphate pathway, the stimulated biosynthesis of abscisic acid and brassinosteroids, and accelerated stress signal transduction [31]. In studies of *Fritillaria cirrhosa* responding to cadmium stress, glutathione metabolism and cell wall biosynthesis pathways were identified as core detoxification mechanisms [32]. The transcriptome profiling of wheat under *Fusarium pseudograminearum* stress further demonstrated that tryptophan metabolism contributes to disease resistance through the accumulation of indole-3-acetaldehyde and melatonin [33]. The molecular interaction mechanisms within the poplar–*B. dothidea* pathosystem remain largely unexplored due to the absence of spatiotemporal transcriptomic data, critically impeding a mechanistic analysis of host–pathogen interactions. Consequently, applying RNA-seq to investigate the poplar–*B. dothidea* interaction will provide a breakthrough approach for systematically dissecting the molecular basis of poplar disease resistance.

We hypothesize that poplar may enhance its disease resistance by activating specific mechanisms upon infection by *B. dothidea*. To test this hypothesis, this study selected *Populus davidiana* × *P. alba* var. *pyramidalis* (Pdpap) as the experimental material. By analyzing morphological traits, physiological and biochemical indicators, and transcriptome data at different time points, we systematically investigated the molecular response mechanisms of Pdpap under *B. dothidea* stress. The objectives of this study were as follows: (a) to elucidate the physiological and biochemical effects of *B. dothidea* stress on Pdpap; (b) to identify key differentially expressed genes (DEGs) responsive to stress and clarify their associated stress-responsive pathways; (c) to screen candidate genes associated with resistance to *B. dothidea* using Weighted Gene Co-expression Network Analysis (WGCNA); and (d) to conduct a comprehensive analysis of the disease-resistant Protein Phosphatase 2C (*PP2C*) family screened by WGCNA. These findings provide novel insights into the molecular mechanisms of poplar resistance against *B. dothidea*, establishing a theoretical foundation for enhancing stress resistance through genetic breeding while identifying critical candidate gene targets.

## 2. Materials and Methods

### 2.1. Biological Materials and Treatment

The experimental plant material was wild-type Pdpap, cultivated on 1/2 MS medium supplemented with 0.01 mg/mL naphthaleneacetic acid (NAA). Two-month-old seedlings with uniform growth were selected for subsequent treatments. Pdpap seedlings were grown in a plant growth chamber at 25 °C (12 h light/12 h dark) until reaching approximately 8 cm in height, then transplanted into an artificial soil mixture (black soil–vermiculite–perlite = 3:1:1) and cultivated in a controlled climate chamber (temperature: 22 ± 2 °C; light intensity: 400 lx/m^2^/s; photoperiod: 16 h light/8 h dark; humidity: 65–75%). The pathogen *B. dothidea* strain was deposited in the China General Microbiological Culture Collection Center (CGMCC 3.27823). The phylogenetic analysis confirming its identity is provided in Figure S1. The strain was cultured on PDA medium in the dark for 7 d until sporulation. During the inoculation procedure, an epidermal incision was made on the stem of Pdpap at 2–3 cm above the soil line. A 6 mm diameter *B. dothidea* mycelial plug was placed on the incision site, while a sterile PDA plug served as the control. Inoculations were performed at 0, 48, 72, 84, 90, and 96 h prior to the unified terminal sampling time (corresponding to treatment durations of 96, 48, 24, 12, 6, and 0 h post-inoculation, respectively; n = 3 plants per group). Subsequently, the second to fourth leaves beneath the apical bud of all plants were simultaneously collected at this final time point. Samples were immediately flash-frozen in liquid nitrogen and stored at −80 °C for subsequent RNA extraction and transcriptome sequencing [34].

### 2.2. RNA Extraction and cDNA Synthesis

Total RNA was extracted using the RNAprep Pure Plant Plus Kit (Tiangen, Beijing, China), and cDNA was synthesized using TransScript First-Strand cDNA Synthesis SuperMix (TransGen, Beijing, China).

### 2.3. Quantitative Real-Time PCR (qPCR) Analysis

To investigate the response of Pdpap−*B. dothidea* treatments, samples were collected at six time points (0, 6, 12, 24, 48, and 96 h) for qRT-PCR analysis. Six pathogenesis-related (PR) protein genes were selected: two defense-related genes (*NPR1* and *PR1*) from the salicylic acid (SA) signaling pathway and four genes (*JAR1*, *COI1*, *JAZ*, and *ORCA3*) from the jasmonic acid (JA) signaling pathway. Primers were designed using Primer 5.0 (sequences listed in Table S1), with *PdpapActin* and *PdPapEF1-α* as internal reference genes. Reactions were performed using a 2× SYBR Green qPCR Master Mix (Bimake, Shanghai, China) on a Stratagene Mx3000P real-time PCR system (Agilent, Santa Clara, CA, USA), following the manufacturer’s protocol. Three technical replicates were included for each gene, and relative gene expression was calculated using the 2^−ΔΔCt^ method [35].

### 2.4. Growth and Physiological Parameter Measurements

To assess the growth response of Pdpap to *B. dothidea* inoculation, plants were wound-inoculated with the pathogen and cultivated in a climate chamber for 20 d. Fresh weight and root length were measured at 0, 5, 10, 15, and 20 d, with three biological replicates per group. Values from Pdpap at 0 d were set as the baseline (normalized to 1). The disease indices of Pdpap were statistically analyzed. Lesion severity was classified into five grades [36]: Grade 0 (healthy/no lesions), Grade 1 (lesion width < 1/3 of stem circumference), Grade 2 (lesion width 1/3–1/2 of stem circumference), Grade 3 (lesion width 1/2–3/4 of stem circumference), and Grade 4 (lesion width > 3/4 of stem circumference or plant mortality). The disease index was calculated using the following formula:

(1) Disease Index (%) = [Σ (Disease grade × Number of plants per grade)/(Total plants surveyed × Maximum disease grade)] × 100

Additionally, samples were collected at 0, 6, 12, 24, and 48 h; flash-frozen in liquid nitrogen; and stored. Peroxidase (POD, Nanjing Jiancheng Bioengineering Institute, Nanjing, China; Cat. No. A084-3-1) and superoxide dismutase (SOD, Cat. No. A001-1-2) activities, malondialdehyde (MDA, Cat. No. A003-3-1) content, and hydrogen peroxide (H_2_O_2_, Cat. No. A064-1-1) levels were quantified using commercial kits according to the manufacturer’s protocols. Three biological replicates were included for each assay [37].

### 2.5. RNA-seq Library Construction and Sequencing

Total RNA was isolated from leaves using the RNeasy Plant Mini Kit (Qiagen, Hilden, Germany) and treated with RNase-free DNase I to remove genomic DNA contamination. RNA integrity was verified by agarose gel electrophoresis, and concentration/quality was assessed using an Agilent 2100 Bioanalyzer. Qualified RNA samples were sent to Personal Biotechnology Co., Ltd. (Shanghai, China) for cDNA library construction and sequencing on the Illumina platform (Illumina, San Diego, CA, USA).

### 2.6. Differential Expression Gene Analysis and Functional Annotation

Raw sequencing data underwent quality assessment, with adapter-containing and low-quality (Q < 20) reads removed. Filtered high-quality reads were aligned to the *Populus trichocarpa* v3.0 reference genome using HISAT2 (http://ccb.jhu.edu/software/hisat2/index.shtml, accessed on 12 January 2024). Gene expression levels were quantified as FPKM (Fragments Per Kilobase of transcript per Million mapped reads) using the Cufflinks package (v2.2.1). Differential expression analysis was performed with DESeq2 (v1.42.0), with DEGs defined as |log_2_(fold change)| ≥ 1 and adjusted *p*-value (*P*adj) ≤ 0.05. Gene Ontology (GO, http://geneontology.org/, accessed on 12 January 2024) enrichment analysis was conducted using topGO (v2.12), and KEGG (http://www.kegg.jp/, accessed on 12 January 2024) pathway enrichment analysis was performed with clusterProfiler (v3.14.3) to identify significantly enriched biological functions and pathways.

### 2.7. qRT-PCR Validation of Gene Expression

To validate the RNA-seq results, 12 randomly selected DEGs were analyzed via qRT-PCR using samples collected at 0, 6, 12, 24, and 48 h. Primers were designed with Primer Premier 6 (sequences in Table S1), and experimental procedures were performed as described in Section 2.3.

### 2.8. WGCNA

Based on RNA-seq-derived gene expression levels (read counts), weighted gene co-expression networks were constructed using the R package WGCNA (v4.1.1). Analyses included module identification, network topology calculation, and module–trait (biochemical indicators) association analysis. Key co-expression networks were visualized using Cytoscape (v3.8.2) [36].

### 2.9. Identification and Characterization of PtrPP2C Gene Family in P. trichocarpa

*P. trichocarpa* genome data was retrieved from Phytozome v12.1 (https://phytozome.jgi.doe.gov/pz/portal.html, accessed on 8 October 2025). Potential *PtrPP2Cs* were screened via whole-genome scanning using HMMER (v3.0). Protein domain integrity was validated through SMART (http://smart.embl-heidelberg.de/, accessed on 8 October 2025) and InterPro (http://www.ebi.ac.uk/interpro/search/sequence, accessed on 8 October 2025), discarding sequences lacking the PP2C domain (PF00481). *Arabidopsis thaliana* PP2C amino acid sequences (TAIR, https://www.arabidopsis.org/, accessed on 8 October 2025) were downloaded for BLASTp (v2.2.28) alignment. Phylogenetic trees were constructed with MEGA v5.1 (Maximum Likelihood, JTT+G+F model) based on Hidden Markov Models (HMMs) from the Pfam database (http://pfam.xfam.org). ProtParam (https://web.expasy.org/protparam/, accessed on 8 October 2025) predicted physicochemical properties (molecular weight, hydrophilicity, etc.), while TBtools (v2.210) visualized gene structures. MEME (http://meme-suite.org/, accessed on 8 October 2025) identified conserved motifs functionally annotated via InterPro (https://www.ebi.ac.uk/interpro/, accessed on 8 October 2025). SOPMA (https://npsa-prabi.ibcp.fr/cgi-bin/npsa_automat.pl?page=npsa_sopma.html, accessed on accessed on 8 October 2025) predicted secondary structures, SWISS-MODEL (https://swissmodel.expasy.org/, accessed on accessed on 8 October 2025) generated tertiary structures, Protter (http://wlab.ethz.ch/protter/start/, accessed on accessed on 8 October 2025) analyzed topological heterogeneity models, and WoLF PSORT (https://wolfpsort.hgc.jp, accessed on accessed on 8 October 2025) forecasted subcellular localization. Promoter sequences (2000 bp upstream) were extracted for cis-regulatory element analysis using PlantCARE (http://bioinformatics.psb.ugent.be/webtools/plantcare/html/, accessed on 8 October 2025), with the results visualized in TBtools. Chromosomal localization and duplication events [MCScanX (Python) for tandem duplications; Dual Synteny Plotter (TBtools, v2.210) for segmental duplications and cross-species synteny with *A. thaliana*, *Eucalyptus grandis*, *Glycine max*, and *Solanum lycopersicum*] were implemented in TBtools. Ka/Ks ratios for duplicated gene pairs were calculated by KaKs_Calculator (v2.0).

### 2.10. qRT-PCR Validation of Key PtrPP2C Genes

To validate the biological functions of the *PtrPP2C* gene family in the Pdpap–*B. dothidea* interaction, qRT-PCR analysis was performed on disease-responsive family members under pathogen stress, with experimental conditions consistent with Section 2.3 and primer sequences provided in Table S1.

### 2.11. Statistical Analysis

Statistical analyses were performed using SPSS 17.0. Intergroup differences were assessed via Student’s *t*-test, with significance set at *p* < 0.05. Different lowercase letters denote significant differences (*p* < 0.05). Data were presented as the mean ± standard error (SE) of three independent biological replicates.

## 3. Results

### 3.1. Morphological and Physiological Responses of Pdpap to B. dothidea Infection

**Figure 1:**
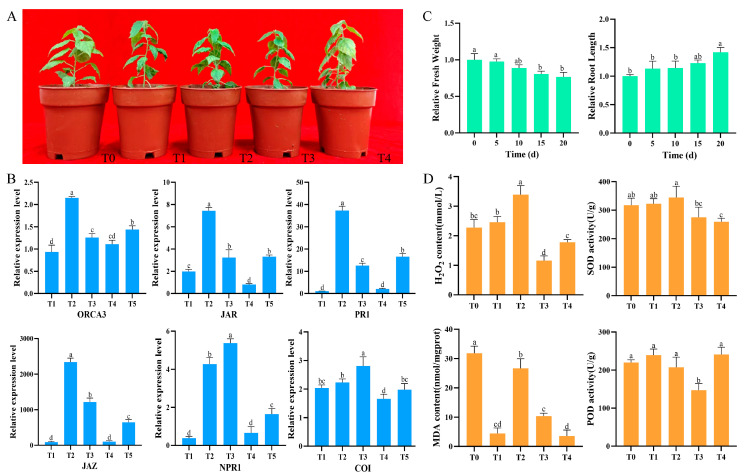
Effects of *Botryosphaeria dothidea* inoculation on morphological and physiological characteristics of *Populus davidiana* × *P. alba* var. *pyramidalis*. (**A**) Plant phenotypic progression. Left to right: Phenotypes at T0 (0 h), T1 (6 h), T2 (12 h), T3 (24 h), T4 (48 h), and T5 (96 h) post-inoculation (three plants per group). (**B**) Expression levels of two defense-related genes (NPR1 and PR1) from salicylic acid signaling pathway and four genes (JAR1, COI1, JAZ, and ORCA3) from jasmonic acid signaling pathway in plants. (**C**) Relative fresh weight and relative root length. (**D**) H_2_O_2_ content, SOD (superoxide dismutase) activity, POD (peroxidase) activity, and MDA (malondialdehyde) content in plant leaves. Error bars indicate standard deviation of three independent biological replicates. Significant differences between groups (*p* < 0.05), determined by Student’s *t*-test, are denoted by different lowercase letters.

To investigate the impact of *B. dothidea* infection on the morphological and physiological characteristics of Pdpap, uniformly grown plants were inoculated with the pathogen for 0, 6, 12, 24, 48, and 96 h, with three biological replicates per treatment group (Figure 1A). Following 96 h of *B. dothidea* inoculation, leaves exhibited chlorosis and progressive abscission. After 10 d of infection, necrotic lesions developed at stem inoculation sites (Figure S2). To establish optimal time points for transcriptome sequencing and physiological assays, a qRT–PCR analysis of six PR genes was performed. The results revealed upregulation of PR gene expression during 0–12 h or 0–24 h, followed by downregulation from 12 to 48 h or 24 to 48 h (Figure 1B). Based on this dynamic expression pattern, plants subjected to *B. dothidea* infection for 0, 6, 12, 24, and 48 h were selected for subsequent transcriptome sequencing and physiological parameter measurements.

To evaluate the impact of *B. dothidea* infection on the growth of Pdpap, changes in fresh weight and root length were measured. The results revealed a significant reduction in fresh weight with infection duration, showing decreases of 2.49%, 11.31%, 19.46%, and 23.53% compared to controls at 5, 10, 15, and 20 d, respectively (Figure 1C). Conversely, root length exhibited sustained growth under stress, increasing by 13.38%, 14.13%, 22.68%, and 42.00% at corresponding time points, indicating a significant suppression of aerial growth but minimal inhibitory effects on root system development. Through a statistical analysis of disease indices, it was found that the disease index of Pdpap reached 83% at 20 d.

To elucidate the response of leaf hormones and reactive oxygen species (ROS) to infection, H_2_O_2_ content, SOD activity, POD activity, and MDA content were quantified (Figure 1D). Significant alterations occurred post-infection versus 0 h: H_2_O_2_ content and SOD activity increased rapidly during early infection, peaking at 12 h before declining progressively; POD activity peaked at 6 h followed by a gradual decrease; MDA content displayed an overall decreasing trend despite a transient peak at 12 h after an increasing tendency during 6–12 h. Collectively, *B. dothidea* infection induces substantial alterations in growth architecture and physiological activity, providing critical insights into host–pathogen interaction mechanisms.

### 3.2. The DEGs of the Transcriptome and Functional Enrichment

To analyze the molecular mechanisms underlying the response of Pdpap to *B. dothidea stress*, transcriptome sequencing was performed on leaves collected at 0, 6, 12, 24, and 48 h. Quality control metrics for all samples are detailed in Table S2, where ‘Sample’ denotes sample identifiers, and ‘Raw_Reads’ represents the number of raw sequencing reads. The average number of raw reads across all treatment samples was approximately 47.61 million. After filtering, an average of 47.07 million high-quality clean reads per sample were obtained. Q20 (Phred score > 20) ranges from 98.83% to 99.05%, where Phred = −10log_10_(e), while Q30 (Phred score > 30) ranges from 96.65% to 97.22%. GC_content reflects the percentage of guanine and cytosine nucleotides in Clean_Reads, ranging from 43.02% to 44.22%. A Principal Component Analysis (PCA) of FPKM values from 15 samples (Figure S3) showed good intragroup clustering and clear intergroup separation, confirming high data quality and reliability for downstream analyses. The raw data was uploaded to the NCBI database (PRJNA1356678).

**Figure 2:**
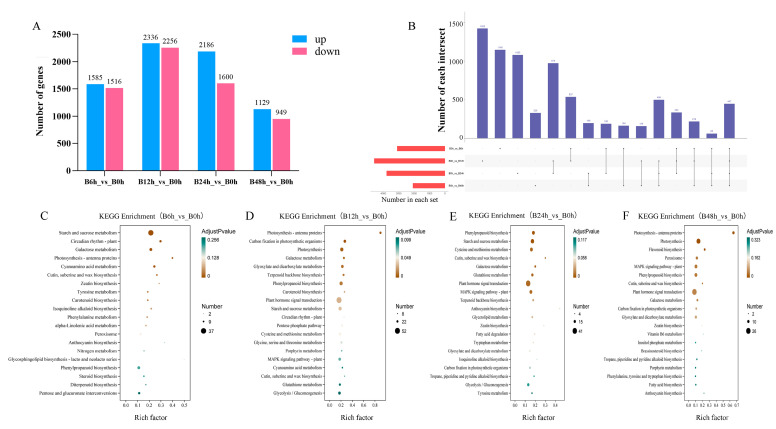
Differentially expressed genes and functional enrichment analysis of *Populus davidiana* × *P. alba* var. *pyramidalis* during *Botryosphaeria dothidea* inoculation. (**A**) Statistics of DEG numbers in different comparison groups. (**B**) UpSet plot illustrating DEG quantities across comparison groups. (**C**–**F**) KEGG pathway enrichment analysis of DEGs (B6h_vs_B0h, B12h_vs_B0h, B24h_vs_B0h, B48h_vs_B0h). Time point notation: B0h (0 h post-inoculation), B6h (6 h), B12h (12 h), B24h (24 h), B48h (48 h). Data represent three biological replicates per group (n = 3), with each replicate corresponding to individual plant.

To investigate changes in gene expression in Pdpap during *B. dothidea* infection, we identified DEGs between treatment groups at different time points (Figure 2A). The results revealed that 3101 DEGs (1585 upregulated, 1516 downregulated) were detected in B6h compared to B0h. Subsequent analyses used B0h as the universal control group. At 12 h, 4592 DEGs (2336 upregulated, 2256 downregulated) were observed. By 24 h, the number of DEGs decreased to 3786 (2186 upregulated, 1600 downregulated), and at 48 h, only 2078 DEGs (1129 upregulated, 949 downregulated) remained. DEG numbers peaked at 12 h, followed by a gradual decline. A further analysis of the DEGs generated an UpSet plot (Figure 2B), demonstrating both shared and exclusive DEGs across treatment groups. Specifically, 447 DEGs were common to all treatment groups (B6h, B12h, B24h, and B48h) compared to B0h, while exclusive DEGs included 1146 (B6h), 1422 (B12h), 1080 (B24h), and 329 (B48h).

To further investigate the functions of DEGs, this study performed GO and KEGG enrichment analyses (Figure S4). The results revealed functional alterations in the host genes of *B. dothidea*-infected PdPap. At 6 h, DEGs were predominantly enriched in molecular functions including transmembrane transporter activity, glucosyltransferase activity, and anion transmembrane transporter activity. By 12 h, enrichment shifted to pigment binding and chlorophyll binding. At 24 h, DEGs significantly concentrated in DNA-binding transcription factor (TF) activity, transcription regulator activity, transcription regulator recruitment activity, and RNA polymerase II transcriptional regulator recruitment activity. Through 48 h, enrichment persistently occurred in molecular function categories such as DNA-binding transcription factor activity and transcription regulator activity. These results collectively indicate that the pathways associated with transcriptional regulation activity were predominantly affected in the plant following pathogen infection.

The functional profiling of DEGs via KEGG enrichment analysis revealed defense-related pathway dynamics in *B dothidea*-infected PdPap (Figure 2C–F). At 6 h post-*B. dothidea* inoculation, defense-related metabolic pathways in PdPap were primarily enriched in carotenoid biosynthesis, phenylalanine metabolism, α-linolenic acid metabolism, phenylpropanoid biosynthesis, diterpenoid biosynthesis, and peroxisome pathway. By 12 h, key enriched pathways shifted to phenylpropanoid biosynthesis, carotenoid biosynthesis, plant hormone signal transduction, and MAPK signaling pathway-plant. During 24–48 h, phenylpropanoid biosynthesis, plant hormone signal transduction, and MAPK signaling pathway-plant maintained persistent enrichment, with flavonoid biosynthesis and peroxisome pathways showing marked induction at 48 h. Crucially, phenylpropanoid biosynthesis (metabolism) exhibited consistent significant enrichment across all infection stages, peaking at 24 h, while plant hormone signal transduction and MAPK signaling pathway-plant (Environmental Information Processing) displayed sustained activation from 12 h to 48 h.

### 3.3. Analysis of Core Disease Resistance Pathways in Transcriptome

To elucidate the molecular mechanisms underlying PdPap resistance to *B. dothidea*, this study focused on three KEGG-identified pathways: plant hormone signal transduction (ko04075), MAPK signaling pathway-plant (ko04016), and phenylpropanoid biosynthesis (ko00940).

**Figure 3:**
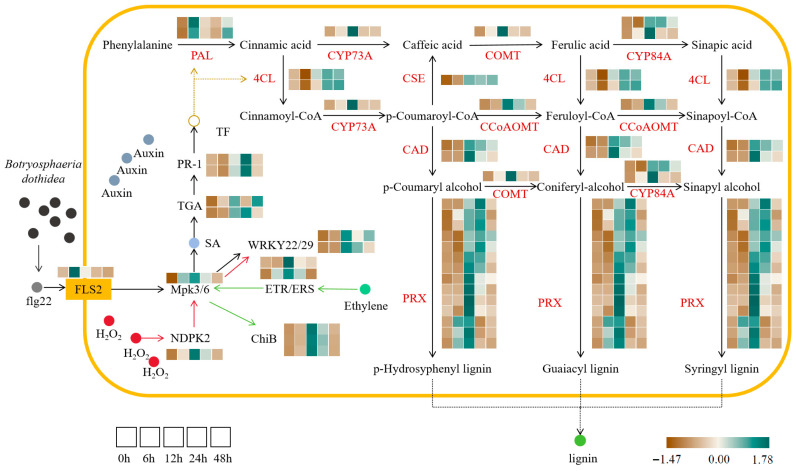
Key pathway analysis in *Populus davidiana* × *P. alba* var. *pyramidalis* across *Botryosphaeria dothidea* inoculation time points. Expression heatmaps depict transcriptional profiles at 0, 6, 12, 24, and 48 h post-inoculation (**left** to **right**). Genes encoding same enzyme are grouped. Expression gradients: brown (low); green (high). Values represent mean normalized expression of differentially expressed genes from three biological replicates (n = 3). PAL: Phenylalanine ammonia-lyase; 4CL: 4-Coumarate-CoA ligase; CYP73A: Cytochrome P450 73A; CSE: Caffeoyl shikimate esterase; CAD: Cinnamyl alcohol dehydrogenase; PRX: Peroxidase; COMT: Caffeic acid O-methyltransferase; CCoAOMT: Caffeoyl-CoA O-methyltransferase; CYP84A: Cytochrome P450 84A.

The MAPK signaling pathway-plant serves as a central hub for biotic stress response in PdPap (Figure 3). Following *B. dothidea* infection, flg22 stimulation activates the pattern recognition receptor FLS2, which is significantly upregulated at 6 h and initiates downstream immune responses; core regulators Mpk3/6 exhibit sustained upregulation (0–12 h), inducing TF WRKY22/29 for early pathogen defense while promoting salicylic acid (SA) synthesis and the concurrent accumulation of the PR1 protein. *B. dothidea*-triggered H_2_O_2_ accumulation enhances NDPK2 (nucleoside diphosphate kinase) expression, activating MPK3/6 via phosphorylation cascades to amplify WRKY22/29 induction. Simultaneously, ethylene signaling is activated: receptors ETR/ERS phosphorylate MPK3/6 upon ethylene perception, thereby upregulating the chitinase gene ChiB, highlighting the pathway’s capacity to integrate multiple defense signals.

Plant hormones constitute a critical defense line against pathogens (Figure 3). DEG analysis revealed the significant upregulation of auxin, ethylene, and SA-related genes following *B. dothidea* infection. Components of the SA pathway, TGA TF and effector protein PR1, displayed an initial increase followed by a decrease pattern (PR1 exhibited transient decline post-inoculation before sustained upregulation), while ethylene receptor ETR showed an identical trend. These activated TFs accelerated phenylpropanoid biosynthesis. KEGG profiling revealed parallel expression trends in phenylpropanoid genes: core lignin synthesis enzymes (PAL, 4CL, CAD), modification enzymes (COMT, CCoAOMT, CYP73A/C4H, CYP84A/F5H, CSE), and peroxidase PRX were significantly induced post-infection, ultimately driving the polymerization of H/G/S-type lignin monomers (p-hydroxyphenyl/guaiacyl/syringyl) to reinforce structural defense in PdPap.

### 3.4. Validation of RNA-seq Results by qPCR

**Figure 4:**
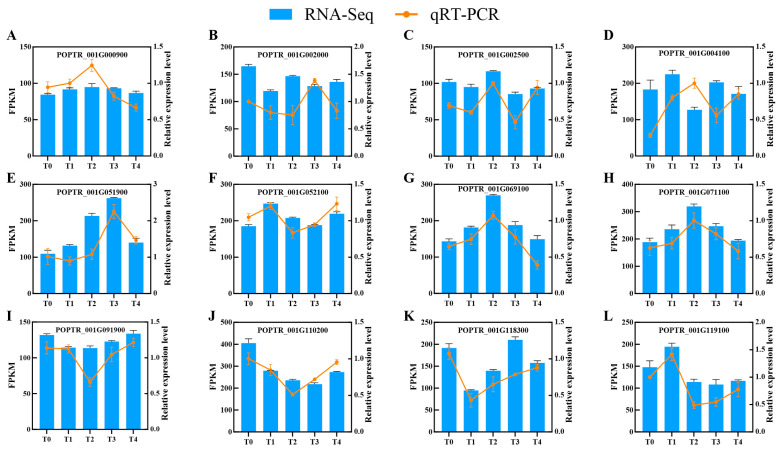
Validation of transcriptome data by RT-qPCR. (A-L): Twelve randomly selected genes from *Botryosphaeria dothidea*-inoculated *Populus davidiana* × *P. alba* var. *pyramidalis* were analyzed. Yellow bars denote RT-qPCR results (fold change); blue bars represent RNA-seq FPKM values. Time points: T0 (0 h), T1 (6 h), T2 (12 h), T3 (24 h), T4 (48 h) post-inoculation (n = 3 plants per group). Error bars indicate mean ± SE from three independent biological replicates.

To verify the accuracy and reproducibility of RNA-seq data, 12 randomly selected DEGs were validated using qPCR. The results demonstrated that the relative expression trends in genes detected by qPCR across *B. dothidea* infection time points were largely consistent with FPKM-based expression patterns from RNA-seq. Minor discrepancies observed in specific genes may arise from technical variations inherent to RNA-seq or qPCR technical variation (Figure 4). Validation experiments confirms the transcriptomic results. 

### 3.5. Identification of Key Disease Resistance Genes in PdPap via WGCNA

**Figure 5:**
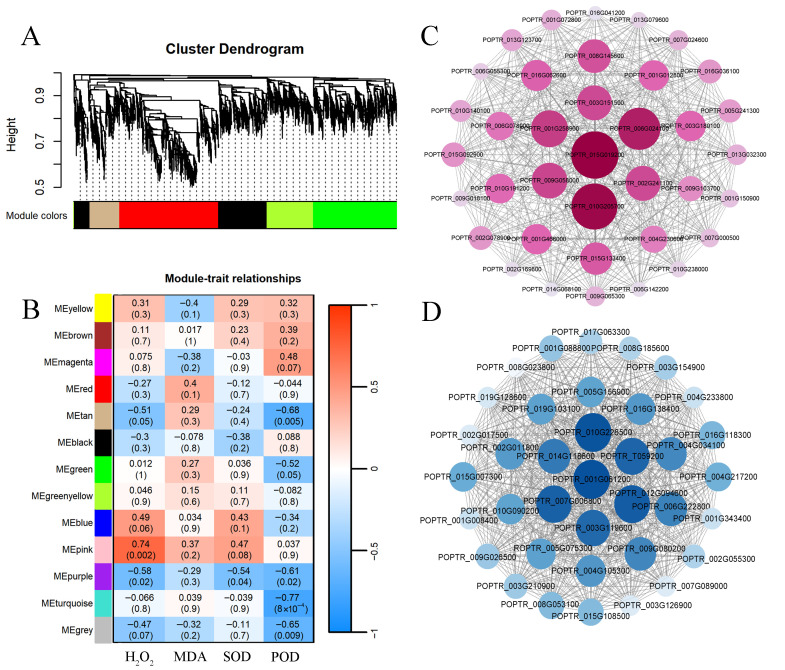
Weighted Gene Co-expression Network Analysis of differentially expressed genes in *Botryosphaeria dothidea*-inoculated *Populus davidiana* × *P. alba* var. *pyramidalis*. (**A**) Cluster dendrogram of DEGs. (**B**) Module–trait relationship heatmap of 13 co-expression modules. (**C**) Hub gene co-expression interaction networks in pink module. (**D**) Hub gene co-expression interaction networks in turquoise module. Data derived from three biological replicates.

Using the physiological indicators of PdPap seedlings (H_2_O_2_ content, MDA content, SOD and POD activity) as trait parameters, combined with gene expression levels across treatment groups, samples were clustered via WGCNA (Figure 5A). Hierarchical clustering dendrograms and trait heatmaps revealed similar gene expression patterns in temporally adjacent samples. Genes with analogous expression profiles were grouped into co-expression modules, resulting in 13 distinct modules labeled by color. A heatmap analysis of module–trait correlations (color depth and numerical values indicate correlation strength; *p*-values denote significance) identified the pink (896 genes) and turquoise (2673 genes) modules as exhibiting the highest correlations (Figure 5B). Applying screening thresholds (Pearson’s |R| > 0.7, *p* < 0.05), the pink and turquoise modules showed significant associations with physiological and biochemical indicators. These results suggest that genes within these modules may modulate the response of PdPap to *B. dothidea* stress. Consequently, the turquoise and pink modules were selected for in-depth analysis.

By constructing an interaction network for the pink and turquoise modules using Cytoscape, 14 hub genes (defined as highly interconnected nodes within the co-expression network) were identified. After sorting these genes by degree (Figure 5C,D), their protein sequences were aligned against the *P. trichocarpa* proteome using InterProScan. The sequences with the highest match were ultimately functionally annotated (Table S3). Notably, the most critical hub genes in the two modules were *P2C76* (POPTR_015G019200, which is designated as *PtrPP2C105* in the subsequent *PtrPP2C* gene family identification and analysis) and *NAC83* (POPTR_001G061200), respectively. For subsequent research, the hub gene *P2C76* from the pink module, which exhibited the highest degree within the positive correlation relationship, was selected as the target of study.

### 3.6. Identification and Characterization of PtrPP2C Family in P. trichocarpa

**Figure 6:**
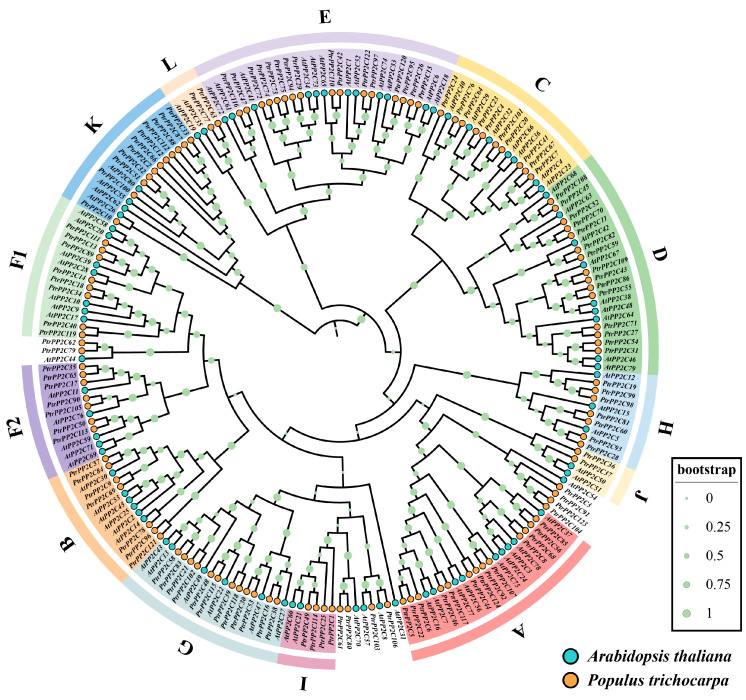
A phylogenetic analysis of Protein Phosphatase 2C genes in *Populus trichocarpa* and *Arabidopsis thaliana*. The maximum likelihood evolutionary tree was constructed using MEGA5 with the JTT+G+F model, analyzing 204 *PP2C* proteins from both species. Distinct subgroups are color-coded, with yellow dots denoting *P. trichocarpa* genes and blue dots representing *A. thaliana* genes.

To elucidate the evolutionary background of *P2C76* and the functional landscape of its gene family in poplar, we systematically identified 124 *PtrPP2C* genes across the genome of the model species *P. trichocarpa*, designated sequentially by genomic position (Table S4). Phylogenetic analysis with *A. thaliana* PP2C homologs classified *PtrPP2Cs* into 13 subgroups of varying sizes (Figure 6): Subgroup E (16 genes) was the largest; Subgroups J and L (each 2 genes) were the smallest; Subgroups A, B, C, D, F1, G, H, I, and K contained 13, 7, 10, 15, 8, 12, 7, 4, and 11 genes, respectively, with *PtrPP2C105* residing in the 7-member F2 subgroup. Notably, the 80 *A. thaliana* PP2C genes were distributed randomly across all subgroups.

Physicochemical property analysis revealed significant divergence among *PtrPP2C* family members, though there was high conservation within subgroups (Table S4). These proteins range from 97 to 909 amino acids (mean = 402) with molecular weights of 10.70–100.76 kDa (mean = 44.21 kDa). Theoretical isoelectric point (pI) distribution showed 95 acidic (pI < 7) and 29 alkaline (pI > 7) proteins, spanning pI 4.42–9.66. Hydrophobicity parameters included aliphatic indices (67.88–101.87) and Grand Average of Hydropathicity (GRAVY) values (−0.59 to 0.15), identifying 121 hydrophilic (GRAVY < 0) and only 1 hydrophobic (GRAVY > 0) protein. Stability analysis indicated 78 unstable proteins (instability index > 40, max = 75.89) and 46 stable proteins (<40), with indices ranging from 28.56 to 75.89.

**Figure 7:**
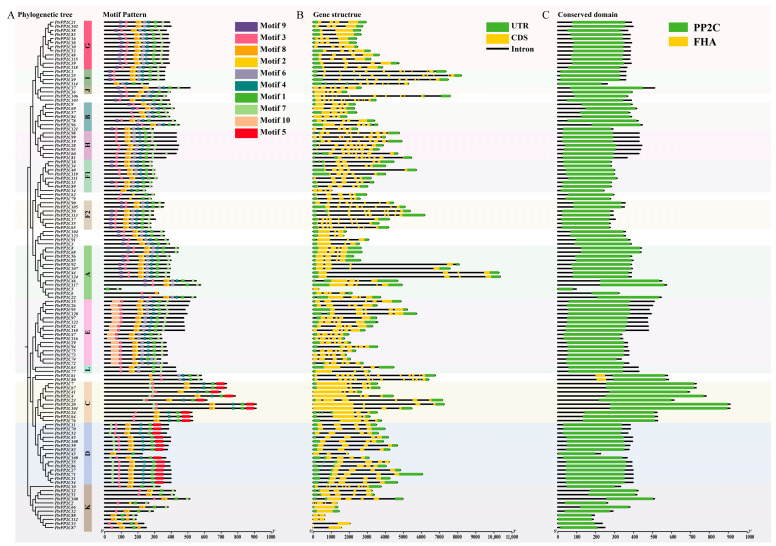
Genomic structural characterization of 124 Protein Phosphatase 2C genes *in Populus trichocarpa*. (**A**) Motif pattern analysis. Different colored boxes represent different motifs. (**B**) Gene structure analysis. Yellow boxes represent coding sequences (CDSs); green boxes indicate untranslated regions (UTRs); black lines denote introns. (**C**) Conserved domain analysis. Subgroup classification of genes was determined by phylogenetic tree analysis.

Ten conserved motifs identified by MEME and annotated via InterProScan (Figure 7A) confirmed Motifs 1/2/3/5/6 as characteristic *PP2C* domains, while five motifs (4/7/8/9/10) remain functionally uncharacterized. All 124 *PtrPP2Cs* contained characteristic motifs, validating family membership. Notably, subgroup-specific motifs were observed: Motif 5 exclusively in Subgroups C/D and Motif 10 solely in Subgroup E. Closely related *PtrPP2Cs* shared similar motif architectures, suggesting that combinatorial motif assembly may drive functional diversification in this family.

Gene structure analysis revealed that *PtrPP2Cs* within the same evolutionary subgroup share similar exon–intron organization, with closely related members exhibiting higher structural conservation (Figure 7B). While significant structural divergence existed among subgroups, phylogenetically adjacent members maintained architectural similarity. Notably, all F2 subgroup members (including *PtrPP2C105*) contained eight exons, and structurally analogous genes (*PtrPP2C90*, *PtrPP2C50*, *PtrPP2C113*, *PtrPP2C17*, *PtrPP2C35*, and *PtrPP2C65*) may collectively function in disease–stress responses. Divergent evolutionary levels among subgroups suggest functional specialization. Conserved domain analysis (Figure 7C) confirmed the universal presence of *PP2C* domains, with *PtrPP2C61* and *PtrPP2C80* additionally harboring FHA domains. Collectively, these findings indicate that functional diversification in the *PP2C* family stems from dynamic molecular evolution, coordinately driven by motif combinations, domain differences, and exon–intron structural divergence.

The secondary structure analysis of PtrPP2Cs (Table S5) revealed α-helices (mean 129.56 residues, 32.78%), extended strands (58.46 residues, 15.10%), and dominant random coils (214.48 residues, 52.13%), collectively demonstrating random coils and α-helices as the primary structural forms. Tertiary structures were modeled using PP2C homologs from diverse species (Table S6), with PtrPP2Cs sharing 85.33% mean sequence identity (70.16~100.00%) with their templates. Topological heterogeneity analysis (Table S7) identified N-glycosylation sites in 116 PtrPP2Cs, signal peptides in 7 members (PtrPP2C6/22/37/61/78/80/96), and transmembrane helices in 8 (PtrPP2C31/36/43/54/55/86/103/108). Subcellular localization (Table S4) showed 49 proteins in chloroplasts, 36 cytoplasmic, 28 nuclear, 5 cytoskeletal, and 4 mitochondrial, with PtrPP2C36 and PtrPP2C106 co-localized to chloroplasts and mitochondria.

**Figure 8:**
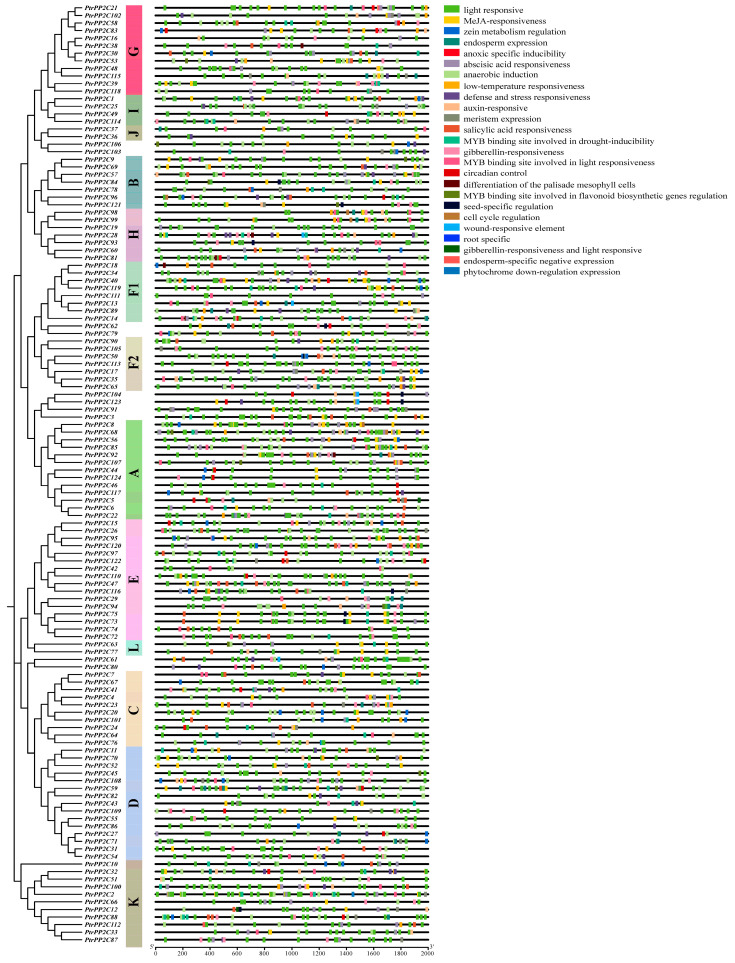
Analysis of cis-acting elements in promoter regions of 124 Protein Phosphatase 2C genes in *Populus trichocarpa*. Different colors indicate distinct types of cis-acting elements. Colored boxes represent specific heatmap modules. Color gradient in heatmap reflects relative abundance of cis-acting elements per gene. Red color lump represents high expression quantity; blue color lump means low expression quantity. Subgroup classification of genes was determined by phylogenetic tree analysis.

The prediction of cis-regulatory elements within the 2000 bp upstream region of *PtrPP2C* start codons using PlantCARE (Figure 8) revealed predominant involvement in stress responses and phytohormone signaling. These elements function in hormonal pathways (e.g., ABA, SA, JA, GA, auxin) and critical biological processes including the following: environmental stress adaptation (drought, low temperature, hypoxia, wounding, pathogens), light responsiveness, endosperm/meristem-specific expression, zein metabolic regulation, circadian control, cell cycle regulation, seed-specific modulation, MYB-binding sites for flavonoid biosynthetic genes, and palisade mesophyll cell differentiation. This diverse distribution suggests that *PtrPP2Cs* may integrate multiple signaling pathways to coordinately regulate plant development and stress adaptation.

PtrPP2Cs were non-uniformly distributed across all 19 chromosomes of *P. trichocarpa* (Figure S5), with no correlation between chromosome size and gene density. Chr10 harbored the highest density (15 genes), followed by Chr1 (14 genes), while Chr4 and Chr17 contained the fewest (2 each). This distribution pattern suggests an association with genomic duplication events.

**Figure 9:**
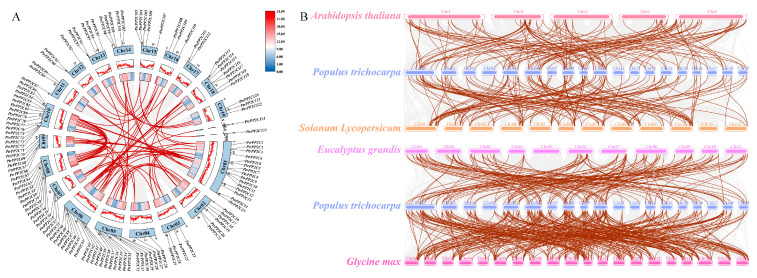
Collinearity analysis of Protein Phosphatase 2C genes in *Populus trichocarpa*. (**A**) Intrachromosomal syntenic relationships of *PtrPP2Cs*. Blue blocks denote chromosomes. Red lines indicate syntenic pairs of *PtrPP2Cs*. Gray lines represent background syntenic references across *P. trichocarpa* genome. Chr1-19 corresponds to chromosomes 1–19. (**B**) Synteny analysis between *PtrPP2Cs* and orthologs from four plant species (*Glycine max*, *Eucalyptus grandis*, *Solanum lycopersicum*, and *Arabidopsis thaliana*). Brown lines connect syntenic *PtrPP2C*–ortholog pairs, while gray lines depict genome-wide orthologous synteny between *P. trichocarpa* and compared species.

MCScanX analysis (Figure 9A) identified 2 tandem duplication pairs on Chr10 (*PtrPP2C72*/*PtrPP2C73* and *PtrPP2C74*/*PtrPP2C75*), alongside 78 segmental duplication pairs involving 99 genes randomly dispersed across chromosomes (Figure 6). Segmental duplications contributed more significantly to *PtrPP2C* diversity than tandem events. Ka/Ks analysis revealed a mean value of 0.19 (range 0–0.64) for segmentally duplicated genes, consistent with purifying selection (Ka/Ks < 1), indicating functional constraints during evolution.

An analysis of syntenic relationships between *PtrPP2Cs* and orthologs from four dicot species (*G. max*, *E. grandis*, *S. lycopersicum*, and *A. thaliana*) (Figure 9B) revealed high cross-species conservation. The results revealed 315 syntenic events between *PtrPP2Cs* and *G. max* orthologs, 149 with *S. lycopersicum*, and 134 with *E. grandis*, whereas only 125 syntenic relationships were retained with *A. thaliana*.

Statistical analysis quantified the number of up- and downregulated DEGs across four treatment groups (Figure S6). The results identified two genes persistently upregulated and eight genes consistently downregulated across all treatments.

**Figure 10:**
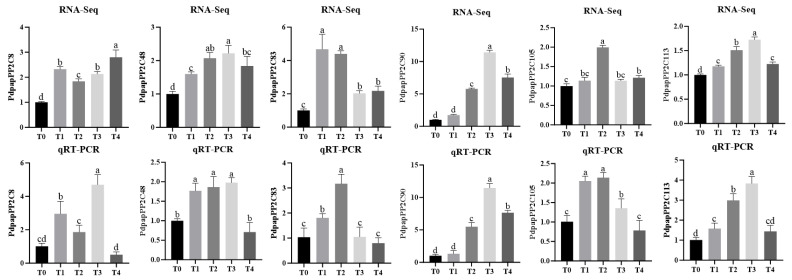
Expression analysis of selected Protein Phosphatase 2C genes in *Populus davidiana* × *P. alba* var. *pyramidalis* via RNA-seq and qRT-PCR. RNA-seq expression was quantified as FPKM. T0, T1, T2, T3, and T4 denote 0, 6, 12, 24, and 48 h post-*Botryosphaeria dothidea* inoculation, respectively. Error bars represent standard errors of three biological replicates (one plant per replicate). Intergroup differences were assessed by Student’s *t*-test with significance threshold of *p* < 0.05; distinct lowercase letters indicate statistically significant differences (*p* < 0.05).

To investigate the functional roles of *PdPapPP2C* genes in poplar, qRT-PCR was performed to measure the expression levels of *PdPapPP2Cs* that were significantly upregulated across ≥3 DEG datasets under *B. dothidea* stress. The results demonstrated consistent expression trends between RNA-seq and qRT-PCR across treatments (Figure 10), though minor discrepancies in some genes may arise from technical variations.

## 4. Discussion

*Populus* spp., serving as ecologically protective, economically productive, and carbon-sequestering broad-leaved trees, are extensively cultivated in temperate regions worldwide. Notably, poplar canker, caused by *B. dothidea*, exhibits a global distribution. This disease induces stem cankers and dieback (particularly devastating in saplings), potentially leading to whole-plant mortality, thereby severely compromising poplar productivity and ecological functions. Consequently, investigating the response mechanisms of poplar to *B. dothidea* is critically important. To address this, this study systematically examined the defense mechanisms of PdPap (resistant cultivar) against *B. dothidea* stress by integrating morphological, physiological, biochemical, and time-series transcriptomic analyses. The findings reveal significant alterations in its antioxidant defense capacity, phytohormone signaling networks, stress signal transduction, and TF regulation.

Specifically, qRT-PCR analysis revealed that 0, 6, 12, 24, and 48 h represent critical time points for PdPap in response to *B. dothidea* infection. Crucially, as physiological phenotypes constitute the terminal manifestation of molecular responses and metabolic alterations, their development inevitably lags behind transcriptional regulation [31]. Consequently, the dynamic response of the transcriptome precedes phenotypic changes induced by *B. dothidea* infection in PdPap; accordingly, these time points (0, 6, 12, 24, and 48 h) were selected for transcriptome sequencing.

Based on this time-series transcriptome analysis, we focused on ROS—pivotal signaling molecules in plant biotic stress responses that play dual roles in maintaining physiological homeostasis and activating defense pathways. Under normal conditions, ROS remain in dynamic equilibrium. However, when subjected to biotic stress, plants experience substantial ROS accumulation. To quantify this oxidative challenge, we monitored key indicators: H_2_O_2_ (reflecting oxidative stress intensity) and MDA (a biomarker of membrane lipid peroxidation), alongside antioxidant enzymes (POD, SOD) that mitigate ROS toxicity [32]. Our data demonstrate that post-inoculation, SOD and POD activities peaked at 6 h or 12 h, which coincided with the maximal H_2_O_2_ accumulation at 12 h attributable to SOD-catalyzed dismutation that generates H_2_O_2_. Notably, while the overall MDA content declined during infection, a transient increase occurred at 6–12 h. This pattern suggests that early *B. dothidea* infection triggers massive ROS accumulation, initially overwhelming antioxidant capacity and exacerbating membrane damage before peroxidase induction restores redox homeostasis. Consequently, MDA returns to baseline levels, confirming the successful containment of oxidative injury [38]. Collectively, these dynamic profiles of antioxidant activity and oxidative markers delineate the plant’s phased defense mobilization against pathogen assault.

In response to *B. dothidea* stress, PdPap establishes a cascade-coordinated defense system involving the MAPK signaling pathway [39], plant hormone signal transduction [40], and phenylpropanoid biosynthesis [41]. The MAPK pathway serves as an early hub within 6 h post-infection by activating the pattern recognition receptor FLS2 via flg22, while ethylene signaling triggers ETR/ERS receptor phosphorylation; these synergistically drive the sustained upregulation of core kinases MPK3/6, with pathogen-induced H_2_O_2_ burst further amplifying MPK3/6 activity through an NDPK2 phosphorylation cascade; activated MPK3/6 induces TF WRKY22/29 to initiate PTI/ETI dual immunity and promotes SA synthesis; additionally, MPK3/6 directly upregulates the chitinase gene ChiB to degrade *B. dothidea*’s cell wall. Simultaneously, MAPK-mediated hormone synthesis interacts with plant hormone signal transduction, upregulating ethylene receptor ETR and SA pathway component TGA-PR1 to activate key phenylpropanoid enzymes (PAL/4CL/CAD), ultimately driving the targeted deposition of H-G-S-type lignin in cell walls; this forms a defense mechanism from cell membrane receptor perception (FLS2/ETR) through MPK3/6-facilitated TF expression accelerating lignin deposition, while the absence of significant JA pathway activation indicates that PdPap primarily relies on an SA-dominated defense strategy against *B. dothidea*.

This study identified pink and turquoise modules through WGCNA, constructing a co-expression network for poplar canker resistance genes based on intramodular connectivity to detect 14 hub genes. The hub gene *P2C76* (*PtrPP2C105*) exhibiting the highest connectivity in positive correlation networks was selected for functional investigation. The *PP2C* gene family plays pivotal roles in ABA and MAPK signaling pathways, serving as essential components in plant environmental adaptation [42]. This finding aligns with the key pathways identified in prior transcriptomic analyses of *B. dothidea* resistance mechanisms in PdPap. Phylogenetic analysis classified 124 *PtrPP2Cs* into 13 subgroups, consistent with PP2C evolutionary classifications in *Litchi chinensis* [43] and *Cucumis sativus* [44]. The expansion of *PtrPP2Cs* was predominantly driven by segmental duplication events (78 pairs, Ka/Ks = 0.19, purifying selection), a mode reflecting woody plants’ evolutionary adaptation to complex environments while circumventing genomic instability from tandem duplication. Topological heterogeneity analysis identified N-glycosylation sites in 116 *PtrPP2Cs*. In contrast to prior research emphasizing *PP2C* functions in abiotic stresses (cold, salt, drought), this work identifies *P2C76* as a disease resistance candidate gene [45,46,47]. Significantly, all F2 subgroup members (including *PtrPP2C105*) share identical gene architectures, motif compositions, and domain organizations. Consequently, *PtrPP2C105* and its structurally homologous counterparts (*PtrPP2C90*, *PtrPP2C50*, *PtrPP2C113*, *PtrPP2C17*, *PtrPP2C35*, and *PtrPP2C65*) may cooperatively modulate disease–stress responses. *PdPapPP2C90*, *PdPapPP2C105*, and *PdPapPP2C113* (all belonging to the F2 subgroup) exhibited significantly positive responses to fungal stress. These genes may serve as core targets for disease resistance studies, providing pivotal candidates for genetic trait improvement and resistant cultivar breeding. Collectively, these findings demonstrate that functional diversification in *PtrPP2Cs* arises through segmental duplication, domain remodeling, and cis-regulatory evolution, providing a theoretical foundation for *PP2C* gene applications in disease-resistant genetic improvement in forest trees.

Although this study revealed the defense mechanisms of PdPap in response to *B. dothidea* stress by integrating physiological, biochemical, and transcriptomic data and screened out core regulatory genes, the molecular interactions within its functional network and the experimental validation of these core genes still require in-depth investigation. Therefore, subsequent research should focus on three key areas: First, research should utilize CRISPR-Cas9-mediated gene editing technology to construct stable knockout and over-expression lines of *PdPapPP2C105* to validate the function of this gene. Second, based on gradient treatments with exogenous hormones, studies should screen for key defense hormones that positively regulate the synthesis of *PdPapPP2C105*. Finally, the yeast two-hybrid screening (Y2H) and co-immunoprecipitation–mass spectrometry (Co-IP-MS) techniques should be integrated to systematically identify the interaction effects between *PdPapPP2C105* and key enzymes in the phenylpropanoid pathway (PAL/4CL/CAD), thereby elucidating the cascade pathway of defense signals from transcriptional regulation to metabolic execution.

## 5. Conclusions

This study established the *B. dothidea*–Pdpap interaction system as the research model. Through time-series transcriptome profiling under *B. dothidea* stress, we deciphered molecular defense mechanisms, revealing the significant upregulation of genes associated with the MAPK signaling pathway, phytohormone transduction, and phenylpropanoid biosynthesis. WGCNA further identified *P2C76* as a hub gene, complemented by a comprehensive analysis of the *PP2C* gene family in *P. trichocarpa*. Collectively, this work delineates the molecular defense framework of PdPap against *B. dothidea*, with the screened key genes serving as breeder-targeted candidates for disease-resistant poplar cultivar development.

## Data Availability

The data presented in this study are openly available in NCBI (https://submit.ncbi.nlm.nih.gov/subs/sra/, accessed on 1 March 2026), PRJNA1356678.

## Supplementary Materials

The following supporting information can be downloaded at https://www.mdpi.com/article/10.3390/jof12010003/s1, Figure S1: Phylogenetic tree constructed using Neighbor-Joining method in MEGA11 based on ITS1/ITS4 sequences; Figure S2: Lesion manifestations in *Populus davidiana* × *P. alba* var. *pyramidalis* stems 10 d post-inoculation with *Botryosphaeria dothidea*; Figure S3: Principal Component Analysis of FPKM values from 15 *Botryosphaeria dothidea*-inoculated *Populus davidiana* × *P. alba* var. *pyramidalis* transcriptome samples; Figure S4: Gene Ontology functional enrichment analysis of *Populus davidiana* × *P. alba* var. *pyramidalis* during *Botryosphaeria dothidea* inoculation; Figure S5: Chromosomal distribution of 124 Protein Phosphatase 2C genes in *Populus trichocarpa*; Figure S6: Venn diagrams of differentially expressed genes in *Populus davidiana* × *P. alba* var. *pyramidalis* under distinct *Botryosphaeria dothidea* treatments. Table S1: Primer sequence; Table S2: Quality control analysis of transcriptome of Pdpap in response to canker infection; Table S3: Genes identified by WGCNA; Table S4: RENAME and physicochemical property analysis; Table S5: Secondary structure of protein; Table S6: Tertiary structure of protein; Table S7: Topological heterogeneity models.
