# Supplementary material for: Integrated Phenotypic, Physiological, Biochemical, and Transcriptomic Analyses Reveal the Molecular Response Mechanisms of Populus to Poplar Canker
Source: J Fungi (Basel). 2025 Dec 20;12(1):3. doi: 10.3390/jof12010003 (PMC12842748; doi:10.3390/jof12010003)
Supplement: Supplementary file 1 [file jof-12-00003-s001.zip › Supplementary Figures.pdf]

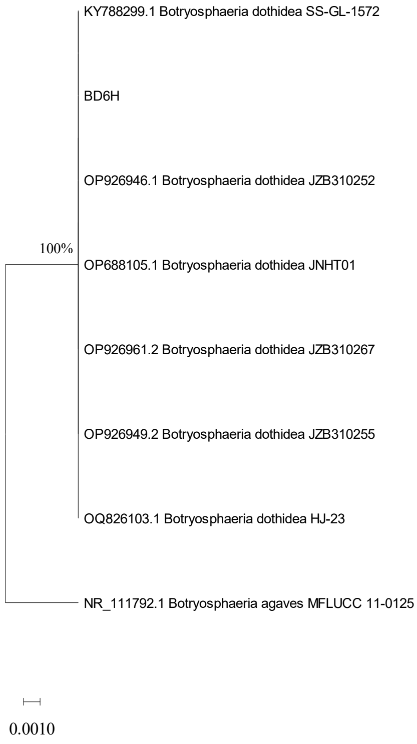

Figure S1. Phylogenetic tree constructed using the Neighbor-Joining method in MEGA11 based on ITS1/ITS4 sequences. The *Botryosphaeria dothidea* strain BD6H used in this study is highlighted.

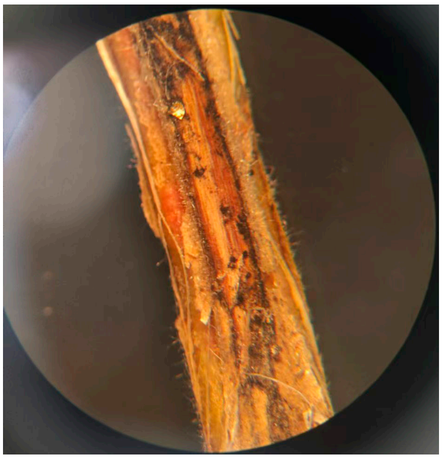

Figure S2. Lesion manifestations in *Populus davidiana* × *P. alba* var. *pyramidalis* stems 10 d post-inoculation with *Botryosphaeria dothidea*.

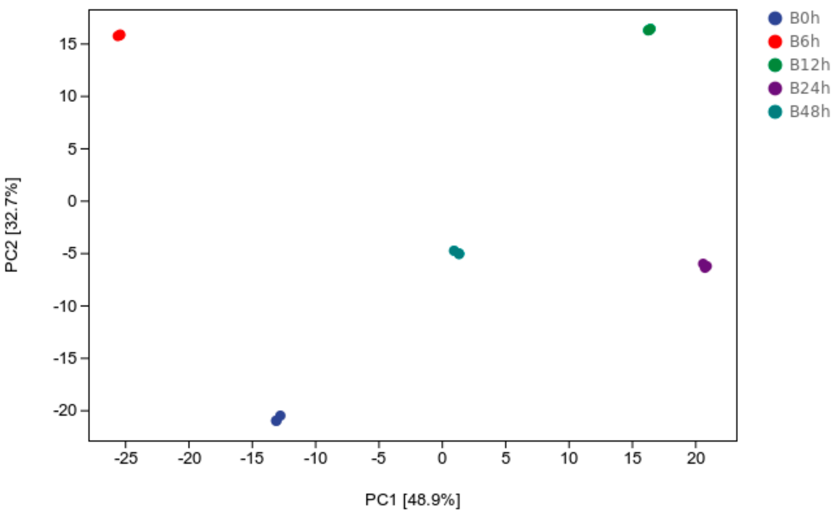

Figure S3. Principal Component Analysis of FPKM values from 15 *Botryosphaeria dothidea*-inoculated *Populus davidiana* × *P. alba* var. *Pyramidalis* transcriptome samples.

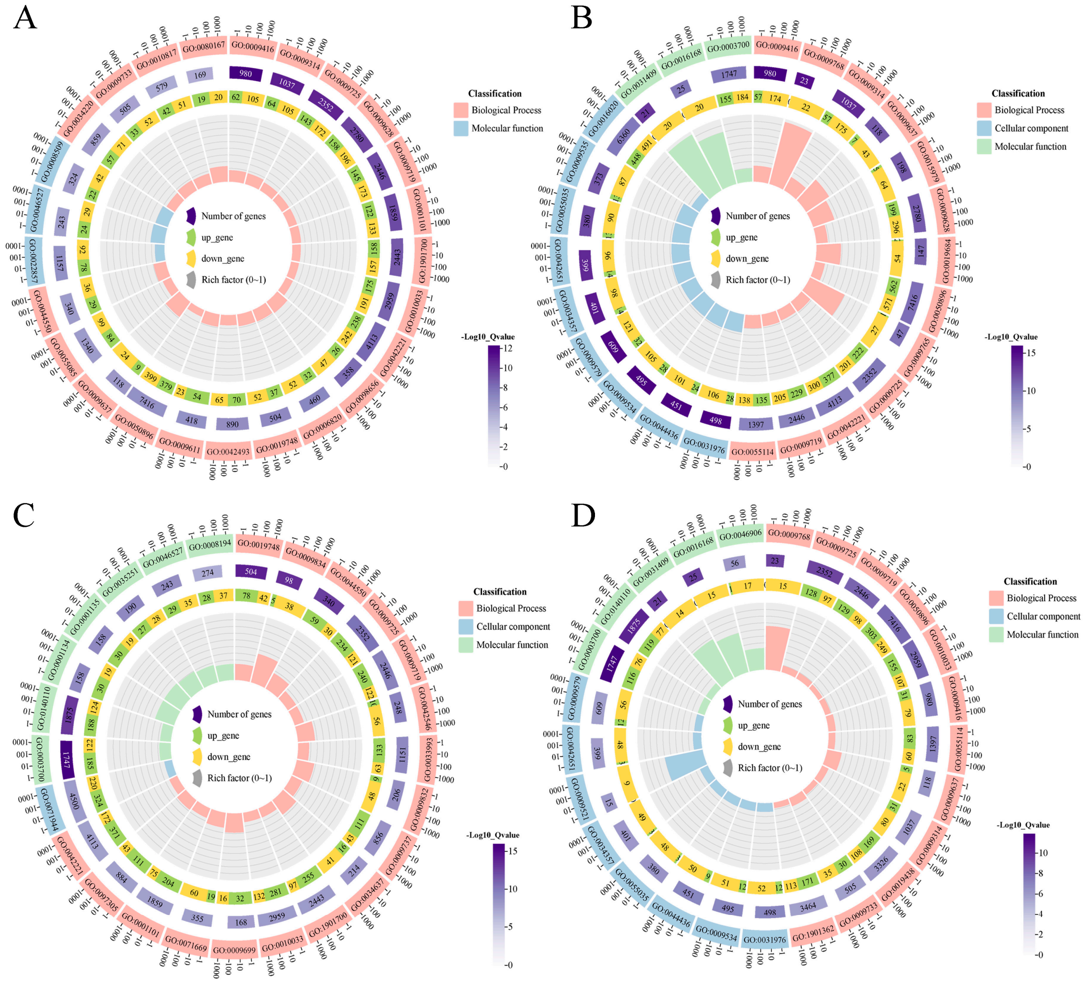

Figure S4. Gene Ontology Functional Enrichment Analysis of *Populus davidiana* × *P. alba* var. *pyramidalis* during *Botryosphaeria dothidea* inoculation. A-D: Gene Ontology enrichment analysis of DEGs

(B6h_vs_B0h, B12h_vs_B0h, B24h_vs_B0h, B48h_vs_B0h). Time point notation: B0h (0 h post-inoculation), B6h (6 h), B12h (12 h), B24h (24 h), B48h (48 h). Data represent three biological replicates per group (n=3), with each replicate corresponding to an individual plant.

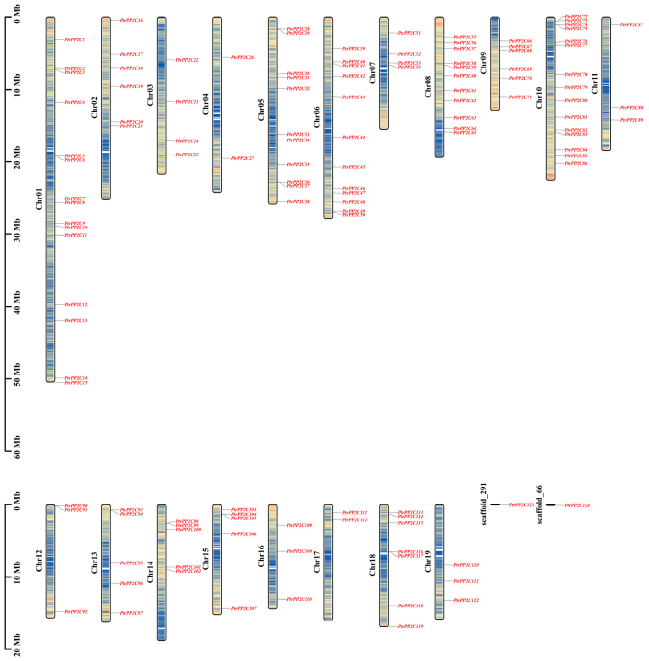

Figure S5. Chromosomal distribution of 124 Protein Phosphatase 2C genes in *Populus trichocarpa*. Chromosomes are labeled as Chr01–Chr19. Scaffold 291 and Scaffold 66 denote anchored genomic regions.

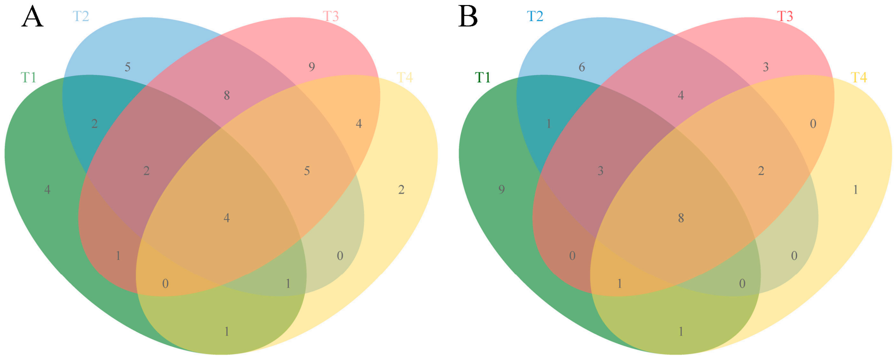

Figure S6. Venn diagrams of Differentially Expressed Genes in *Populus davidiana* × *P. alba* var. *Pyramidalis* under Distinct *Botryosphaeria dothidea* treatments. A: Up-regulated DEGs; B: Down-regulated DEGs. Time point notation: B0h (0 h post-inoculation), B6h (6 h), B12h (12 h), B24h (24 h), B48h (48 h).
