# Supplementary material for: Integrated Phenotypic, Physiological, Biochemical, and Transcriptomic Analyses Reveal the Molecular Response Mechanisms of Populus to Poplar Canker
Source: J Fungi (Basel). 2025 Dec 20;12(1):3. doi: 10.3390/jof12010003 (PMC12842748; doi:10.3390/jof12010003)
Supplement: Supplementary file 1 [file jof-12-00003-s001.zip › Table S1 Primer sequence.pdf]

Table S1. Primer sequences

Name	Forward primer	Reverse primer
NPR1	GGCCGACGATACTTCCCTAGTT	TGCCCTCATAGTTTCCTGAGCT
PR1	ACACCACCGTGCAAGCCTATG	CGAGCAGAGTTACGCCAAACCA
JAR1	AGTGGTGAACCAAGCGAGGAG	GGATTTGCTGCACCTTGCTGTT
COI	GAGGTACTTGTGGGTGCATGGT	ACGCAGAACCTACCCTCGCT
JAZ	GGAAGCTCCATTGGCACAGATG	CCGGAGTTGGGTTGTTTGTCTG
ORCA3	AGAGGAGTGAGGCAAAGACCCT	TCCGGCTCTCGACGCTTAGT
Pdpapactin	GCTGAGAGATTCCGTTGCCCTG	GGCGGTGATCTCCTTGCTCATT
PdpapEF1-α	TGGGTCGTGTTGAAACTGGTGT	GGCAGGATCGTCCTTGGAGTTC
POPTR_001G000900	CTGTTGCTGCGATATGGT	TTGATGATGAGGATGGTGAG
POPTR_001G002000	AGAGTGTGCCATCAAGTG	CATCATCCTCGCTGTAAGA
POPTR_001G002500	GGCTGCTGAACAAGACTAT	CCACCAACCACAATACCTT
POPTR_001G004100	TTGATGGTGGTCTTGATGG	GCAAGAGCAACAAGAGATG
POPTR_001G051900	ACCTCCGTCCTTCTTCTT	GCAACAATCACAACATCATC
POPTR_001G052100	TAATGGATTGAAGCCGATTG	TCACAGGAGGAGTCAGTAA
POPTR_001G069100	CGTCTTGTTCAGTCTCCAA	TCACTCGCTCTTCTTCCTA
POPTR_001G071100	CGCTATTGGTGTCGTCAT	CTCATCATTCTTCTTCTTCCTC
POPTR_001G091900	GTCCTGTGTATCGTAGTCTT	TTACCAGCCTTGCCATTC
POPTR_001G110200	CTCACTGCTGCTCTAACC	CATAATGGCGAGATTCTGTC
POPTR_001G118300	TAACCAGAGCGAGCAATG	CGTGATGGAAGCCTAATGT
POPTR_001G119100	AGGCAACGATTGAACCAT	TGTCTTCCATCTCCTCAGT
PP2C8	TTACCGTGGAGTAGTGAGT	CGAATCTGAGCCGTCTAAT
PP2C48	ATGCTACCATCTGCCAAG	TGCCACAACTAACAACCT

PP2C85	TGACCACAGACGAAGAGT	GCACGACAATCACCAGTA
PP2C93	GAGGAGGTGGTGAAGGAT	TCAGGTCTGTCAGGCTTAT
PP2C107	TGGAGAGTAGGAGGTGTATT	TCTTCTTCTGTTCTGGCAAT
PP2C115	TTGCTCGTCGCTAATGTT	TCTGGTCTGGCTTGTGAT
