# Supplementary material for: Integrated Phenotypic, Physiological, Biochemical, and Transcriptomic Analyses Reveal the Molecular Response Mechanisms of Populus to Poplar Canker
Source: J Fungi (Basel). 2025 Dec 20;12(1):3. doi: 10.3390/jof12010003 (PMC12842748; doi:10.3390/jof12010003)
Supplement: Supplementary file 1 [file jof-12-00003-s001.zip › Table S2. Quality control analysis of the transcriptome of Pdpap in response to canker infection.pdf]

Table S2. Quality control analysis of the transcriptome of Pdpap in response to canker infection.

Sample	Raw_Reads	Clean_Reads	Clean_Bases	Q20	Q30	GC_content
B0h_1	52,066,632	51,504,412	7,750,366,831	98.97	96.99	43.99
B0h_2	44,957,962	44,446,512	6,701,871,642	98.95	96.93	43.89
B0h_3	44,543,844	44,062,402	6,644,721,373	98.98	96.98	43.86
B6h_1	46,755,100	46,199,658	6,968,002,010	98.94	96.90	44.19
B6h_2	46,388,894	45,874,832	6,916,914,404	98.93	96.75	44.25
B6h_3	47,881,158	47,334,426	7,132,392,278	98.94	96.91	44.28
B12h_1	44,176,688	43,677,836	6,582,091,588	98.94	96.89	43.59
B12h_2	45,679,754	45,156,550	6,801,081,509	98.92	96.86	43.60
B12h_3	48,000,824	47,409,396	7,139,710,648	98.89	96.79	43.62
B24h_1	49,010,662	48,447,780	7,295,402,757	98.92	96.89	43.59
B24h_2	53,105,370	52,616,180	7,919,057,061	99.05	97.22	43.62
B24h_3	51,126,032	50,569,566	7,609,635,142	98.94	96.95	43.59
B48h_1	56,226,130	55,592,310	8,381,117,383	98.92	96.85	43.65
B48h_2	35,784,270	35,340,998	5,327,931,932	98.85	96.67	43.60
B48h_3	48,447,846	47,816,792	7,203,356,334	98.83	96.65	43.53
