# Supplementary material for: Integrated Phenotypic, Physiological, Biochemical, and Transcriptomic Analyses Reveal the Molecular Response Mechanisms of Populus to Poplar Canker
Source: J Fungi (Basel). 2025 Dec 20;12(1):3. doi: 10.3390/jof12010003 (PMC12842748; doi:10.3390/jof12010003)
Supplement: Supplementary file 1 [file jof-12-00003-s001.zip › Table S5 Secondary structure of protein.pdf]

Table S5. Predition of the Sencondary Structure in PtrPP2C Proteins

Name in this paper	Locus tag	Number of Alpha helix	The percent of all the sencondary structures	Number of Extended strand	The percent of all the sencondary structures	Number of Random coil	The percent of all the sencondary structures
PtrPP2C1	Potri.001G043000v3.0	141	39.28%	60	16.71%	158	44.01%
PtrPP2C2	Potri.001G089200v3.0	68	25.86%	46	17.49%	149	56.65%
PtrPP2C3	Potri.001G092100v3.0	123	31.70%	52	13.40%	213	54.90%
PtrPP2C4	Potri.001G144700v3.0	215	27.46%	100	12.77%	468	59.77%
PtrPP2C5	Potri.001G198400v3.0	39	40.21%	17	17.53%	41	42.27%
PtrPP2C6	Potri.001G198500v3.0	83	25.70%	11	3.41%	229	70.90%
PtrPP2C7	Potri.001G239300v3.0	239	32.74%	77	10.55%	414	56.71%
PtrPP2C8	Potri.001G245200v3.0	132	29.93%	51	11.56%	258	58.50%
PtrPP2C9	Potri.001G278500v3.0	108	27.55%	57	14.54%	227	57.91%
PtrPP2C10	Potri.001G282500v3.0	92	27.79%	55	16.62%	184	55.59%
PtrPP2C11	Potri.001G297200v3.0	125	32.72%	63	16.49%	194	50.79%
PtrPP2C12	Potri.001G381000v3.0	77	26.46%	52	17.87%	162	55.67%
PtrPP2C13	Potri.001G398100v3.0	106	37.59%	58	20.57%	118	41.84%
PtrPP2C14	Potri.001G465200v3.0	88	36.07%	52	21.31%	104	42.62%
PtrPP2C15	Potri.001G473300v3.0	138	27.11%	67	13.16%	304	59.72%
PtrPP2C16	Potri.002G007500v3.0	142	37.37%	52	13.68%	186	48.95%
PtrPP2C17	Potri.002G074500v3.0	107	35.31%	53	17.49%	143	47.19%
PtrPP2C18	Potri.002G097200v3.0	99	35.87%	50	18.12%	127	46.01%
PtrPP2C19	Potri.002G127300v3.0	138	32.17%	59	13.75%	232	54.08%
PtrPP2C20	Potri.002G185000v3.0	230	25.30%	92	10.12%	587	64.58%
PtrPP2C21	Potri.002G190400v3.0	153	39.33%	54	13.88%	182	46.79%
PtrPP2C22	Potri.003G044200v3.0	170	31.14%	58	10.62%	318	58.24%
PtrPP2C23	Potri.003G089500v3.0	166	27.08%	72	11.75%	375	61.17%
PtrPP2C24	Potri.003G159600v3.0	160	30.59%	72	13.77%	291	55.64%
PtrPP2C25	Potri.003G183800v3.0	145	40.39%	59	16.43%	155	43.18%
PtrPP2C26	Potri.004G066200v3.0	142	28.40%	65	13.00%	293	58.60%
PtrPP2C27	Potri.004G177100v3.0	133	33.50%	52	13.10%	212	53.40%
PtrPP2C28	Potri.005G021200v3.0	114	25.73%	62	14.00%	267	60.27%
PtrPP2C29	Potri.005G021900v3.0	111	30.16%	63	17.12%	194	52.72%
PtrPP2C30	Potri.005G102500v3.0	152	39.90%	57	14.96%	172	45.14%
PtrPP2C31	Potri.005G108500v3.0	132	33.25%	58	14.61%	207	52.14%
PtrPP2C32	Potri.005G125700v3.0	107	25.30%	64	15.13%	252	59.57%
PtrPP2C33	Potri.005G160600v3.0	73	31.20%	46	19.66%	115	49.15%
PtrPP2C34	Potri.005G164600v3.0	100	35.34%	55	19.43%	128	45.23%
PtrPP2C35	Potri.005G186000v3.0	102	36.96%	58	21.01%	116	42.03%
PtrPP2C36	Potri.005G214500v3.0	133	33.93%	59	15.05%	200	51.02%

PtrPP2C37	Potri.005G214700v3.0	179	35.03%	72	14.09%	260	50.88%
PtrPP2C38	Potri.005G253700v3.0	136	34.96%	51	13.11%	202	51.93%
PtrPP2C39	Potri.006G059600v3.0	122	31.52%	52	13.44%	213	55.04%
PtrPP2C40	Potri.006G081400v3.0	122	40.67%	55	18.33%	123	41.00%
PtrPP2C41	Potri.006G085000v3.0	202	29.06%	76	10.94%	417	60.00%
PtrPP2C42	Potri.006G105000v3.0	148	31.03%	62	13.00%	267	55.97%
PtrPP2C43	Potri.006G134100v3.0	96	42.67%	39	17.33%	90	40.00%
PtrPP2C44	Potri.006G164600v3.0	122	31.44%	53	13.66%	213	54.90%
PtrPP2C45	Potri.006G192600v3.0	140	35.53%	54	13.71%	200	50.76%
PtrPP2C46	Potri.006G224600v3.0	156	28.47%	60	10.95%	332	60.58%
PtrPP2C47	Potri.006G232700v3.0	110	32.64%	59	17.51%	168	49.85%
PtrPP2C48	Potri.006G248400v3.0	125	32.72%	55	14.40%	202	52.88%
PtrPP2C49	Potri.006G265100v3.0	145	40.50%	59	16.48%	154	43.02%
PtrPP2C50	Potri.006G267600v3.0	109	37.33%	54	18.49%	129	44.18%
PtrPP2C51	Potri.007G028900v3.0	89	21.34%	60	14.39%	268	64.27%
PtrPP2C52	Potri.007G051900v3.0	115	31.08%	57	15.41%	198	53.51%
PtrPP2C53	Potri.007G058700v3.0	135	35.16%	52	13.54%	197	51.30%
PtrPP2C54	Potri.007G061100v3.0	130	32.75%	51	12.85%	216	54.41%
PtrPP2C55	Potri.008G046900v3.0	135	34.53%	53	13.55%	203	51.92%
PtrPP2C56	Potri.008G059200v3.0	119	30.51%	52	13.33%	219	56.15%
PtrPP2C57	Potri.008G070400v3.0	110	28.95%	62	16.32%	208	54.74%
PtrPP2C58	Potri.008G100700v3.0	151	40.92%	59	15.99%	159	43.09%
PtrPP2C59	Potri.008G104300v3.0	136	35.79%	53	13.95%	191	50.26%
PtrPP2C60	Potri.008G123600v3.0	146	34.11%	67	15.65%	215	50.23%
PtrPP2C61	Potri.008G149700v3.0	170	29.36%	88	15.20%	321	55.44%
PtrPP2C62	Potri.008G168400v3.0	108	36.49%	57	19.26%	131	44.26%
PtrPP2C63	Potri.008G198700v3.0	85	20.05%	71	16.75%	268	63.21%
PtrPP2C64	Potri.008G207700v3.0	166	31.56%	73	13.88%	287	54.56%
PtrPP2C65	Potri.008G209000v3.0	102	36.96%	58	21.01%	116	42.03%
PtrPP2C66	Potri.009G021300v3.0	106	27.89%	53	13.95%	221	58.16%
PtrPP2C67	Potri.009G030600v3.0	234	32.05%	72	9.86%	424	58.08%
PtrPP2C68	Potri.009G037300v3.0	137	31.21%	52	11.85%	250	56.95%
PtrPP2C69	Potri.009G073000v3.0	116	27.88%	55	13.22%	245	58.89%
PtrPP2C70	Potri.009G091600v3.0	129	33.68%	60	15.67%	194	50.65%
PtrPP2C71	Potri.009G137400v3.0	132	33.25%	59	14.86%	206	51.89%
PtrPP2C72	Potri.010G006100v3.0	141	37.60%	65	17.33%	169	45.07%
PtrPP2C73	Potri.010G006200v3.0	119	31.73%	64	17.07%	192	51.20%
PtrPP2C74	Potri.010G009100v3.0	125	37.31%	48	14.33%	162	48.36%
PtrPP2C75	Potri.010G009200v3.0	119	31.73%	64	17.07%	192	51.20%

PtrPP2C76	Potri.010G024800v3.0	211	40.04%	68	12.90%	248	47.06%
PtrPP2C77	Potri.010G028300v3.0	117	27.34%	67	15.65%	244	57.01%
PtrPP2C78	Potri.010G047600v3.0	140	33.02%	51	12.03%	233	54.95%
PtrPP2C79	Potri.010G070100v3.0	100	35.84%	46	16.49%	133	47.67%
PtrPP2C80	Potri.010G091500v3.0	138	23.63%	100	17.12%	346	59.25%
PtrPP2C81	Potri.010G121600v3.0	125	34.15%	57	15.57%	184	50.27%
PtrPP2C82	Potri.010G146700v3.0	125	33.24%	57	15.16%	194	51.60%
PtrPP2C83	Potri.010G151500v3.0	163	42.67%	57	14.92%	162	42.41%
PtrPP2C84	Potri.010G187000v3.0	108	27.76%	54	13.88%	227	58.35%
PtrPP2C85	Potri.010G199600v3.0	101	25.44%	56	14.11%	240	60.45%
PtrPP2C86	Potri.010G214700v3.0	138	35.29%	52	13.30%	201	51.41%
PtrPP2C87	Potri.011G013000v3.0	75	30.24%	44	17.74%	129	52.02%
PtrPP2C88	Potri.011G102200v3.0	67	34.72%	34	17.62%	92	47.67%
PtrPP2C89	Potri.011G116700v3.0	113	40.07%	61	21.63%	108	38.30%
PtrPP2C90	Potri.012G002100v3.0	111	31.27%	58	16.34%	186	52.39%
PtrPP2C91	Potri.012G002700v3.0	132	34.65%	53	13.91%	196	51.44%
PtrPP2C92	Potri.012G131800v3.0	133	34.19%	53	13.62%	203	52.19%
PtrPP2C93	Potri.013G011600v3.0	117	26.77%	73	16.70%	247	56.52%
PtrPP2C94	Potri.013G012200v3.0	115	31.25%	66	17.93%	187	50.82%
PtrPP2C95	Potri.013G085500v3.0	148	30.71%	65	13.49%	269	55.81%
PtrPP2C96	Potri.013G099400v3.0	146	32.74%	66	14.80%	234	52.47%
PtrPP2C97	Potri.013G144100v3.0	137	28.96%	63	13.32%	273	57.72%
PtrPP2C98	Potri.014G031200v3.0	145	33.80%	63	14.69%	221	51.52%
PtrPP2C99	Potri.014G031500v3.0	145	33.80%	65	15.15%	219	51.05%
PtrPP2C100	Potri.014G042800v3.0	121	23.73%	67	13.14%	322	63.14%
PtrPP2C101	Potri.014G110500v3.0	270	29.74%	75	8.26%	563	62.00%
PtrPP2C102	Potri.014G115500v3.0	153	38.54%	60	15.11%	184	46.35%
PtrPP2C103	Potri.015G010600v3.0	132	34.02%	57	14.69%	199	51.29%
PtrPP2C104	Potri.015G018800v3.0	117	32.87%	63	17.70%	176	49.44%
PtrPP2C105	Potri.015G019200v3.0	144	41.03%	53	15.10%	154	43.87%
PtrPP2C106	Potri.015G043000v3.0	142	38.48%	55	14.91%	172	46.61%
PtrPP2C107	Potri.015G133900v3.0	140	35.62%	55	13.99%	198	50.38%
PtrPP2C108	Potri.016G045600v3.0	128	32.49%	53	13.45%	213	54.06%
PtrPP2C109	Potri.016G082800v3.0	137	37.43%	53	14.48%	176	48.09%
PtrPP2C110	Potri.016G127900v3.0	143	29.92%	65	13.60%	270	56.49%
PtrPP2C111	Potri.017G013300v3.0	96	30.67%	57	18.21%	160	51.12%
PtrPP2C112	Potri.017G023900v3.0	71	37.97%	34	18.18%	82	43.85%
PtrPP2C113	Potri.018G013900v3.0	105	35.96%	57	19.52%	130	44.52%
PtrPP2C114	Potri.018G017800v3.0	94	36.02%	42	16.09%	125	47.89%

PtrPP2C115	Potri.018G033000v3.0	138	36.32%	52	13.68%	190	50.00%
PtrPP2C116	Potri.018G059800v3.0	98	28.65%	66	19.30%	178	52.05%
PtrPP2C117	Potri.018G060300v3.0	169	29.44%	62	10.80%	343	59.76%
PtrPP2C118	Potri.018G115100v3.0	135	37.09%	54	14.84%	175	48.08%
PtrPP2C119	Potri.018G150800v3.0	102	34.11%	63	21.07%	134	44.82%
PtrPP2C120	Potri.019G054200v3.0	159	32.25%	65	13.18%	269	54.56%
PtrPP2C121	Potri.019G071600v3.0	101	34.71%	54	18.56%	136	46.74%
PtrPP2C122	Potri.019G103100v3.0	131	27.64%	58	12.24%	285	60.13%
PtrPP2C123	Potri.T137100v3.0	123	34.45%	60	16.81%	174	48.74%
PtrPP2C124	Potri.T063000v3.0	117	30.15%	56	14.43%	215	55.41%
