# Supplementary material for: Integrated Phenotypic, Physiological, Biochemical, and Transcriptomic Analyses Reveal the Molecular Response Mechanisms of Populus to Poplar Canker
Source: J Fungi (Basel). 2025 Dec 20;12(1):3. doi: 10.3390/jof12010003 (PMC12842748; doi:10.3390/jof12010003)
Supplement: Supplementary file 1 [file jof-12-00003-s001.zip › Table S6 Tertiary structure of protein.pdf]

Table S6. Predition of the protein structure homology-modelling in PtrPP2Cs

Name in this paper	Locus tag	Template	Sequence Identity	Description	Homology-modelling
PtrPP2C1	Potri.001G043000v3.0	A0A2K2B944.1.A	87.74%	protein-serine/threonine phosphatase	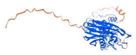
PtrPP2C2	Potri.001G089200v3.0	A0A4U5PLD4.1.A	95.92%	Protein phosphatase	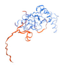
PtrPP2C3	Potri.001G092100v3.0	A0A2K2BUV8.1.A	100.00%	protein-serine/threonine phosphatase	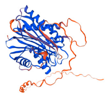
PtrPP2C4	Potri.001G144700v3.0	A0A5N5NCM5.1.A	92.16%	PPM-type phosphatase domain-containing protein	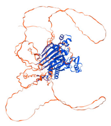
PtrPP2C5	Potri.001G198400v3.0	A0A498J4M0.1.A	82.29%	protein-serine/threonine phosphatase	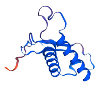
PtrPP2C6	Potri.001G198500v3.0	A0A6A6NEM6.1.A	70.19%	protein-serine/threonine phosphatase	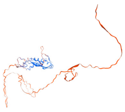
PtrPP2C7	Potri.001G239300v3.0	I1LQQ6.1.A	78.32%	protein-serine/threonine phosphatase	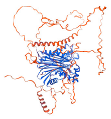
PtrPP2C8	Potri.001G245200v3.0	A0A6M2F478.1.A	97.27%	protein-serine/threonine phosphatase	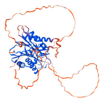
PtrPP2C9	Potri.001G278500v3.0	I1MBC7.1.A	71.35%	protein-serine/threonine phosphatase	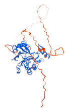
PtrPP2C10	Potri.001G282500v3.0	A0A4U5NPA0.1.A	96.37%	Protein phosphatase	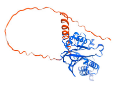

PtrPP2C11	Potri.001G297200v3.0	A0A6M2EC88.1.A	91.36%	protein-serine/threonine phosphatase	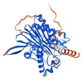
PtrPP2C12	Potri.001G381000v3.0	A0A6M2EAA2.1.A	96.91%	Protein phosphatase	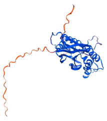
PtrPP2C13	Potri.001G398100v3.0	A0A833Y667.1.A	83.33%	protein-serine/threonine phosphatase	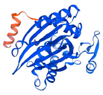
PtrPP2C14	Potri.001G465200v3.0	A0A2K2CE57.1.A	100.00%	PPM-type phosphatase domain-containing protein	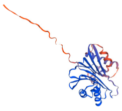
PtrPP2C15	Potri.001G473300v3.0	A0A5E4G7Q4.1.A	70.16%	protein-serine/threonine phosphatase	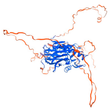
PtrPP2C16	Potri.002G007500v3.0	A0A836IVW0.1.A	88.59%	Uncharacterized protein	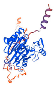
PtrPP2C17	Potri.002G074500v3.0	A9PIA5.1.A	99.67%	protein-serine/threonine phosphatase	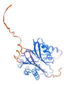
PtrPP2C18	Potri.002G097200v3.0	B9GUW5.1.A	100.00%	protein-serine/threonine phosphatase	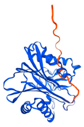
PtrPP2C19	Potri.002G127300v3.0	A0A6J1IQN8.1.A	82.48%	protein-serine/threonine phosphatase	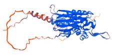
PtrPP2C20	Potri.002G185000v3.0	I1JQY9.1.A	75.28%	PPM-type phosphatase domain-containing protein	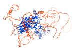
PtrPP2C21	Potri.002G190400v3.0	B9GSY2.1.A	100.00%	protein-serine/threonine phosphatase	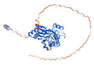

PtrPP2C22	Potri.003G044200v3.0	A0A6A6NEM6.1.A	77.98%	protein-serine/threonine phosphatase	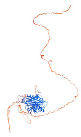
PtrPP2C23	Potri.003G089500v3.0	A0A6P5TMP0.1.A	79.04%	Protein phosphatase 2C 29	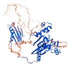
PtrPP2C24	Potri.003G159600v3.0	A0A6M2EWF3.1.A	96.56%	protein-serine/threonine phosphatase	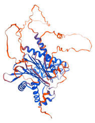
PtrPP2C25	Potri.003G183800v3.0	A0A2K2B944.1.A	100.00%	protein-serine/threonine phosphatase	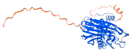
PtrPP2C26	Potri.004G066200v3.0	V4UIQ6.1.A	70.55%	protein-serine/threonine phosphatase	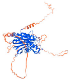
PtrPP2C27	Potri.004G177100v3.0	I1LF55.1.A	86.33%	protein-serine/threonine phosphatase	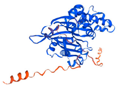
PtrPP2C28	Potri.005G021200v3.0	A0A5B6ZCR0.1.A	86.21%	protein-serine/threonine phosphatase	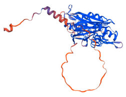
PtrPP2C29	Potri.005G021900v3.0	A0A837A2W6.1.A	86.96%	Uncharacterized protein	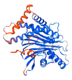
PtrPP2C30	Potri.005G102500v3.0	A0A7J7DXZ1.1.A	75.73%	protein-serine/threonine phosphatase	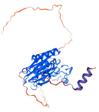
PtrPP2C31	Potri.005G108500v3.0	I1JCD8.1.A	86.01%	protein-serine/threonine phosphatase	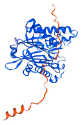
PtrPP2C32	Potri.005G125700v3.0	A0A5N5MPF1.1.A	91.96%	Protein phosphatase	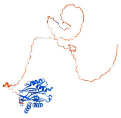

PtrPP2C33	Potri.005G160600v3.0	A0A6M2EAA2.1.A	84.40%	Protein phosphatase	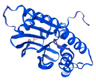
PtrPP2C34	Potri.005G164600v3.0	B9GUW5.1.A	96.11%	protein-serine/threonine phosphatase	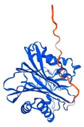
PtrPP2C35	Potri.005G186000v3.0	A0A2P2KA63.1.A	88.77%	protein-serine/threonine phosphatase	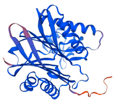
PtrPP2C36	Potri.005G214500v3.0	A0A2K2AK93.1.A	100.00%	protein-serine/threonine phosphatase	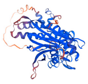
PtrPP2C37	Potri.005G214700v3.0	F6HBI1.1.A	72.10%	PPM-type phosphatase domain-containing protein	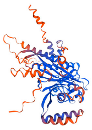
PtrPP2C38	Potri.005G253700v3.0	A0A836IVW0.1.A	95.37%	Uncharacterized protein	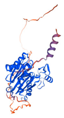
PtrPP2C39	Potri.006G059600v3.0	I1LZW3.1.A	76.24%	protein-serine/threonine phosphatase	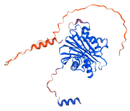
PtrPP2C40	Potri.006G081400v3.0	A0A200R9D8.1.A	78.52%	protein-serine/threonine phosphatase	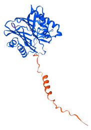
PtrPP2C41	Potri.006G085000v3.0	A0A4U5QGL6.1.A	96.68%	protein-serine/threonine phosphatase	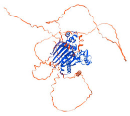
PtrPP2C42	Potri.006G105000v3.0	A0A4U5QAV6.1.A	80.17%	PPM-type phosphatase domain-containing protein	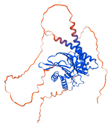
PtrPP2C43	Potri.006G134100v3.0	B9SGT9.1.A	72.56%	protein-serine/threonine phosphatase	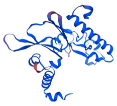

PtrPP2C44	Potri.006G164600v3.0	A0A1S2YFC2.1.A	72.25%	protein-serine/threonine phosphatase	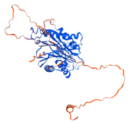
PtrPP2C45	Potri.006G192600v3.0	A0A5N5JKM3.1.A	90.61%	protein-serine/threonine phosphatase	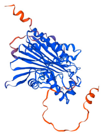
PtrPP2C46	Potri.006G224600v3.0	G1E8X0.1.A	100.00%	protein-serine/threonine phosphatase	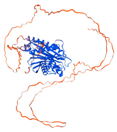
PtrPP2C47	Potri.006G232700v3.0	A0A5C7HXT6.1.A	70.18%	PPM-type phosphatase domain-containing protein	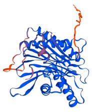
PtrPP2C48	Potri.006G248400v3.0	A0A4U5QR83.1.A	96.34%	protein-serine/threonine phosphatase	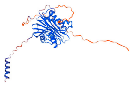
PtrPP2C49	Potri.006G265100v3.0	A0A5B6Z0A5.1.A	84.92%	protein-serine/threonine phosphatase	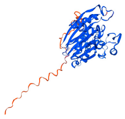
PtrPP2C50	Potri.006G267600v3.0	A0A0R0GC70.1.A	92.78%	protein-serine/threonine phosphatase	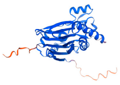
PtrPP2C51	Potri.007G028900v3.0	A0A5N5MPF1.1.A	86.33%	Protein phosphatase	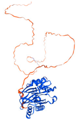
PtrPP2C52	Potri.007G051900v3.0	A0A6P5YZF7.1.A	74.39%	protein-serine/threonine phosphatase	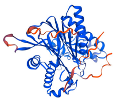
PtrPP2C53	Potri.007G058700v3.0	A0A7J7DXZ1.1.A	74.67%	protein-serine/threonine phosphatase	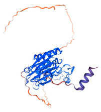
PtrPP2C54	Potri.007G061100v3.0	I1JCD8.1.A	83.21%	protein-serine/threonine phosphatase	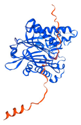

PtrPP2C55	Potri.008G046900v3.0	A0A6P3ZFG4.1.A	84.24%	protein-serine/threonine phosphatase	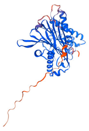
PtrPP2C56	Potri.008G059200v3.0	A0A0R0HTU9.1.A	71.89%	protein-serine/threonine phosphatase	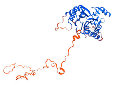
PtrPP2C57	Potri.008G070400v3.0	A0A4U5MX20.1.A	85.79%	protein-serine/threonine phosphatase	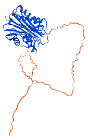
PtrPP2C58	Potri.008G100700v3.0	B9HIJ4.1.A	100.00%	protein-serine/threonine phosphatase	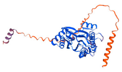
PtrPP2C59	Potri.008G104300v3.0	A0A834SNF1.1.A	83.78%	protein-serine/threonine phosphatase	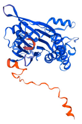
PtrPP2C60	Potri.008G123600v3.0	I1NFM6.1.A	86.21%	protein-serine/threonine phosphatase	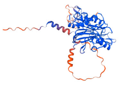
PtrPP2C61	Potri.008G149700v3.0	V4RGN6.1.A	71.88%	protein-serine/threonine phosphatase	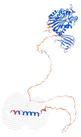
PtrPP2C62	Potri.008G168400v3.0	A0A6M2EKR1.1.A	94.93%	PPM-type phosphatase domain-containing protein	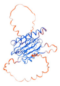
PtrPP2C63	Potri.008G198700v3.0	A0A2K3N917.1.A	70.41%	protein-serine/threonine phosphatase	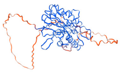
PtrPP2C64	Potri.008G207700v3.0	A0A6P5YLG9.1.A	73.00%	protein-serine/threonine phosphatase	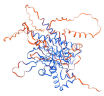
PtrPP2C65	Potri.008G209000v3.0	A0A2P2KA63.1.A	88.77%	protein-serine/threonine phosphatase	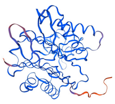
PtrPP2C66	Potri.009G021300v3.0	A0A2K1Z1G6.1.A	100.00%	Protein phosphatase	

					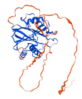
PtrPP2C67	Potri.009G030600v3.0	A0A6N2L6M1.1.A	95.07%	PROTEIN PHOSPHATASE 2C	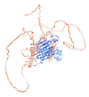
PtrPP2C68	Potri.009G037300v3.0	A0A6M2F410.1.A	97.27%	protein-serine/threonine phosphatase	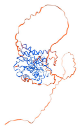
PtrPP2C69	Potri.009G073000v3.0	A0A2K1Z4A3.1.A	100.00%	protein-serine/threonine phosphatase	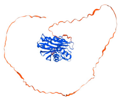
PtrPP2C70	Potri.009G091600v3.0	A0A6M2EC88.1.A	98.69%	protein-serine/threonine phosphatase	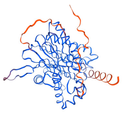
PtrPP2C71	Potri.009G137400v3.0	A0A1U8AT70.1.A	86.87%	protein-serine/threonine phosphatase	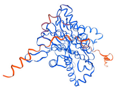
PtrPP2C72	Potri.010G006100v3.0	B9HXE6.1.A	100.00%	protein-serine/threonine phosphatase	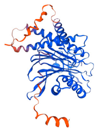
PtrPP2C73	Potri.010G006200v3.0	A0A061ENH6.1.A	79.89%	protein-serine/threonine phosphatase	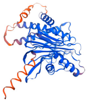
PtrPP2C74	Potri.010G009100v3.0	B9HXE6.1.A	100.00%	protein-serine/threonine phosphatase	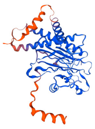
PtrPP2C75	Potri.010G009200v3.0	A0A061ENH6.1.A	79.89%	protein-serine/threonine phosphatase	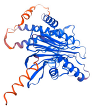
PtrPP2C76	Potri.010G024800v3.0	A0A6P5YLG9.1.A	72.43%	protein-serine/threonine phosphatase	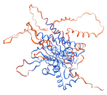

PtrPP2C77	Potri.010G028300v3.0	A0A2K3N917.1.A	72.55%	protein-serine/threonine phosphatase	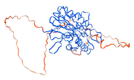
PtrPP2C78	Potri.010G047600v3.0	A0A7J7BYL8.1.A	70.67%	protein-serine/threonine phosphatase	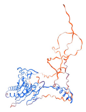
PtrPP2C79	Potri.010G070100v3.0	A0A6P5X540.1.A	70.25%	PPM-type phosphatase domain-containing protein	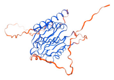
PtrPP2C80	Potri.010G091500v3.0	A0A2I4G9B4.1.A	71.30%	Protein phosphatase 2C 70	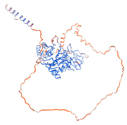
PtrPP2C81	Potri.010G121600v3.0	I1NFM6.1.A	86.46%	protein-serine/threonine phosphatase	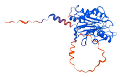
PtrPP2C82	Potri.010G146700v3.0	A0A834SNF1.1.A	83.51%	protein-serine/threonine phosphatase	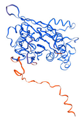
PtrPP2C83	Potri.010G151500v3.0	A0A5N5HBW9.1.A	71.77%	protein-serine/threonine phosphatase	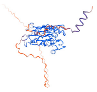
PtrPP2C84	Potri.010G187000v3.0	A0A4U5MX20.1.A	97.69%	protein-serine/threonine phosphatase	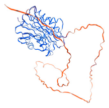
PtrPP2C85	Potri.010G199600v3.0	A0A2C9VM50.1.A	75.51%	protein-serine/threonine phosphatase	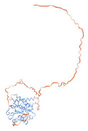
PtrPP2C86	Potri.010G214700v3.0	A0A6P3ZFG4.1.A	82.95%	protein-serine/threonine phosphatase	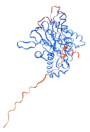
PtrPP2C87	Potri.011G013000v3.0	A0A836J9K5.1.A	79.64%	Uncharacterized protein	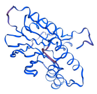

PtrPP2C88	Potri.011G102200v3.0	A0A6M2EAA2.1.A	84.04%	Protein phosphatase	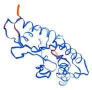
PtrPP2C89	Potri.011G116700v3.0	K7KWZ7.1.A	79.43%	protein-serine/threonine phosphatase	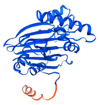
PtrPP2C90	Potri.012G002100v3.0	A0A5N5K1H3.1.A	87.14%	protein-serine/threonine phosphatase	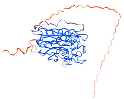
PtrPP2C91	Potri.012G002700v3.0	A0A4U5NA28.1.A	97.11%	protein-serine/threonine phosphatase	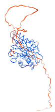
PtrPP2C92	Potri.012G131800v3.0	A0A836Y0F9.1.A	93.06%	Uncharacterized protein	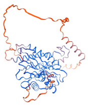
PtrPP2C93	Potri.013G011600v3.0	A0A1Q3CZV2.1.A	84.31%	protein-serine/threonine phosphatase	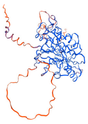
PtrPP2C94	Potri.013G012200v3.0	I1LB78.1.A	78.95%	protein-serine/threonine phosphatase	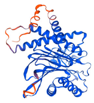
PtrPP2C95	Potri.013G085500v3.0	B9RM68.1.A	86.51%	protein-serine/threonine phosphatase	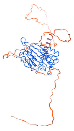
PtrPP2C96	Potri.013G099400v3.0	U5FV08.1.A	100.00%	PPM-type phosphatase domain-containing protein	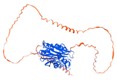
PtrPP2C97	Potri.013G144100v3.0	I1LRZ4.1.A	85.07%	protein-serine/threonine phosphatase	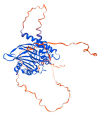
PtrPP2C98	Potri.014G031200v3.0	A0A6J1IQN8.1.A	82.71%	protein-serine/threonine phosphatase	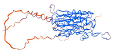

PtrPP2C99	Potri.014G031500v3.0	A0A6J1IQN8.1.A	82.48%	protein-serine/threonine phosphatase	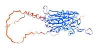
PtrPP2C100	Potri.014G042800v3.0	A0A5J5B2X7.1.A	73.57%	Protein phosphatase	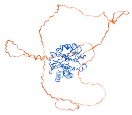
PtrPP2C101	Potri.014G110500v3.0	I1JQY9.1.A	74.57%	PPM-type phosphatase domain-containing protein	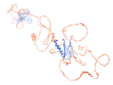
PtrPP2C102	Potri.014G115500v3.0	I1JBC4.1.A	79.12%	protein-serine/threonine phosphatase	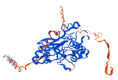
PtrPP2C103	Potri.015G010600v3.0	A0A2I0K6J3.1.A	71.50%	protein-serine/threonine phosphatase	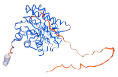
PtrPP2C104	Potri.015G018800v3.0	A0A6N2LXU7.1.A	88.98%	protein-serine/threonine phosphatase	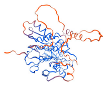
PtrPP2C105	Potri.015G019200v3.0	A0A5N5K1H3.1.A	93.73%	protein-serine/threonine phosphatase	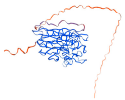
PtrPP2C106	Potri.015G043000v3.0	A0A2K1XHV4.1.A	100.00%	protein-serine/threonine phosphatase	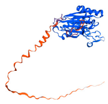
PtrPP2C107	Potri.015G133900v3.0	A0A836Y0F9.1.A	79.64%	Uncharacterized protein	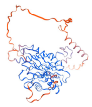
PtrPP2C108	Potri.016G045600v3.0	A0A5N5JKM3.1.A	94.67%	protein-serine/threonine phosphatase	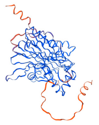
PtrPP2C109	Potri.016G082800v3.0	A0A2H5Q806.1.A	72.33%	protein-serine/threonine phosphatase	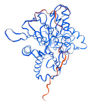

PtrPP2C110	Potri.016G127900v3.0	A0A4U5QAV6.1.A	95.40%	PPM-type phosphatase domain-containing protein	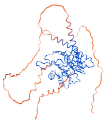
PtrPP2C111	Potri.017G013300v3.0	A0A3N7G3P7.1.A	100.00%	protein-serine/threonine phosphatase	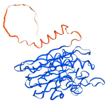
PtrPP2C112	Potri.017G023900v3.0	A0A2K2CA90.1.A	84.86%	Protein phosphatase	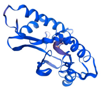
PtrPP2C113	Potri.018G013900v3.0	A0A0R0GC70.1.A	92.44%	protein-serine/threonine phosphatase	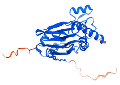
PtrPP2C114	Potri.018G017800v3.0	A0A5B6Z0A5.1.A	82.76%	protein-serine/threonine phosphatase	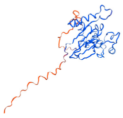
PtrPP2C115	Potri.018G033000v3.0	A0A4U5QR83.1.A	91.05%	protein-serine/threonine phosphatase	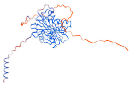
PtrPP2C116	Potri.018G059800v3.0	A0A2C9UAP7.1.A	75.38%	PPM-type phosphatase domain-containing protein	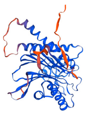
PtrPP2C117	Potri.018G060300v3.0	G1E8X0.1.A	88.69%	protein-serine/threonine phosphatase	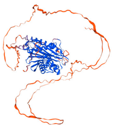
PtrPP2C118	Potri.018G115100v3.0	I1LZW3.1.A	77.16%	protein-serine/threonine phosphatase	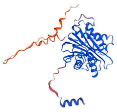
PtrPP2C119	Potri.018G150800v3.0	A0A2N9GMQ0.1.A	79.73%	protein-serine/threonine phosphatase	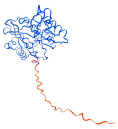
PtrPP2C120	Potri.019G054200v3.0	I1N3Z6.1.A	74.27%	protein-serine/threonine phosphatase	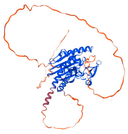

PtrPP2C121	Potri.019G071600v3.0	A0A5J5AAK2.1.A	74.39%	PPM-type phosphatase domain-containing protein	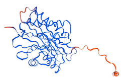
PtrPP2C122	Potri.019G103100v3.0	I1LRZ4.1.A	84.83%	protein-serine/threonine phosphatase	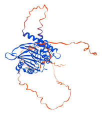
PtrPP2C123	Potri.T137100v3.0	A0A6N2LXU7.1.A	89.55%	protein-serine/threonine phosphatase	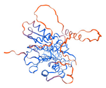
PtrPP2C124	Potri.T063000v3.0	I1K5Y7.1.A	74.80%	protein-serine/threonine phosphatase	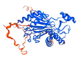
