# Supplementary material for: Integrated Phenotypic, Physiological, Biochemical, and Transcriptomic Analyses Reveal the Molecular Response Mechanisms of Populus to Poplar Canker
Source: J Fungi (Basel). 2025 Dec 20;12(1):3. doi: 10.3390/jof12010003 (PMC12842748; doi:10.3390/jof12010003)
Supplement: Supplementary file 1 [file jof-12-00003-s001.zip › Table S7 Topological heterogeneity models.pdf]

Table S7. The result of the topological heterogeneity model prediction

Name in this paper	Locus tag	Number of N-glycosylation sites	Signal peptides	Topological heterogeneity model
PtrPP2C1	Potri.001G043000v3.0	3	No	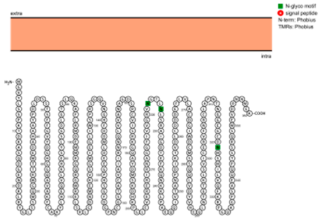
PtrPP2C2	Potri.001G089200v3.0	2	No	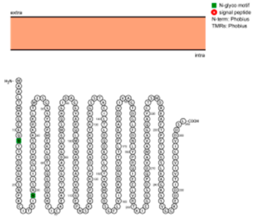
PtrPP2C3	Potri.001G092100v3.0	5	No	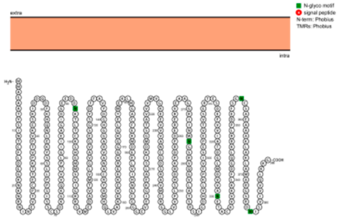
PtrPP2C4	Potri.001G144700v3.0	5	No	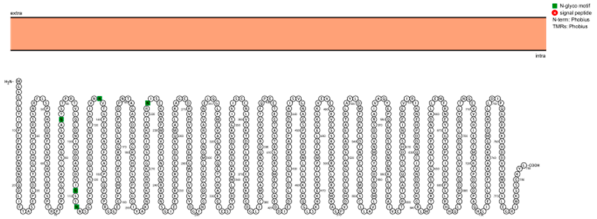

PtrPP2C5	Potri.001G198400v3.0	1	No	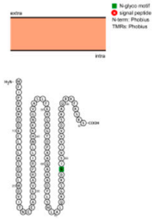
PtrPP2C6	Potri.001G198500v3.0	4	Yes	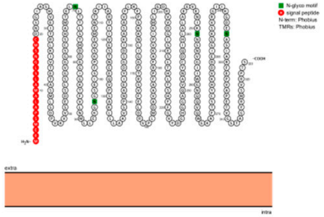
PtrPP2C7	Potri.001G239300v3.0	3	No	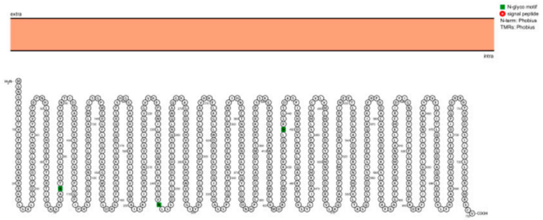
PtrPP2C8	Potri.001G245200v3.0	4	No	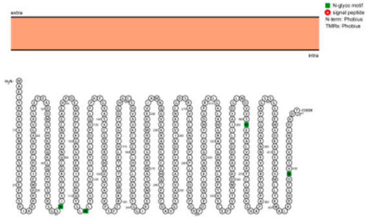

PtrPP2C9	Potri.001G278500v3.0	0	No	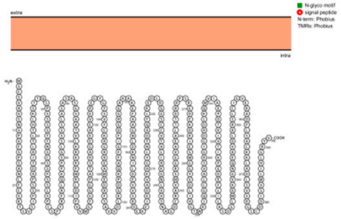
PtrPP2C10	Potri.001G282500v3.0	2	No	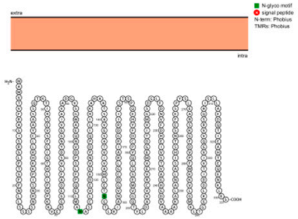
PtrPP2C11	Potri.001G297200v3.0	1	No	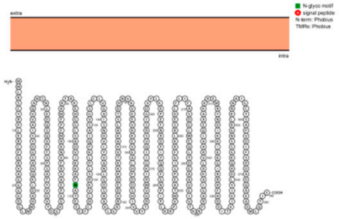
PtrPP2C12	Potri.001G381000v3.0	1	No	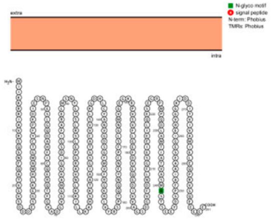

PtrPP2C13	Potri.001G398100v3.0	0	No	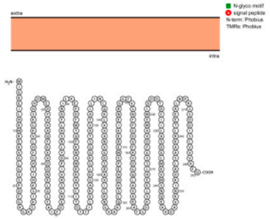
PtrPP2C14	Potri.001G465200v3.0	1	No	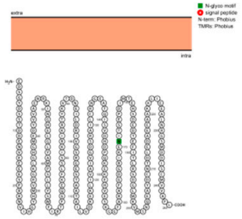
PtrPP2C15	Potri.001G473300v3.0	4	No	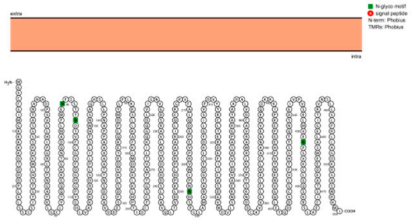
PtrPP2C16	Potri.002G007500v3.0	5	No	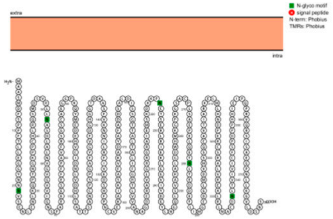

PtrPP2C17	Potri.002G074500v3.0	3	No	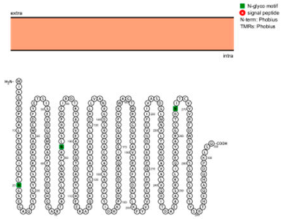
PtrPP2C18	Potri.002G097200v3.0	3	No	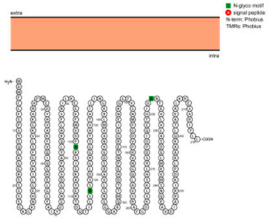
PtrPP2C19	Potri.002G127300v3.0	1	No	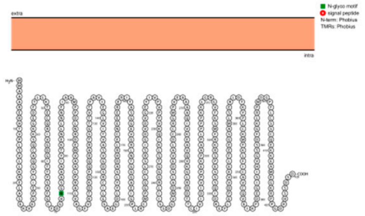
PtrPP2C20	Potri.002G185000v3.0	8	No	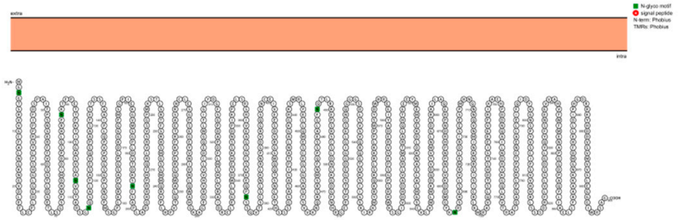

PtrPP2C21	Potri.002G190400v3.0	1	No	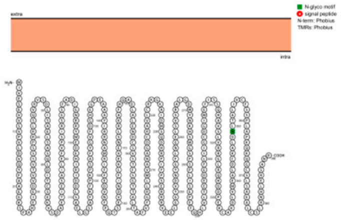
PtrPP2C22	Potri.003G044200v3.0	5	Yes	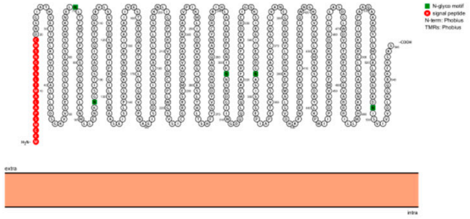
PtrPP2C23	Potri.003G089500v3.0	3	No	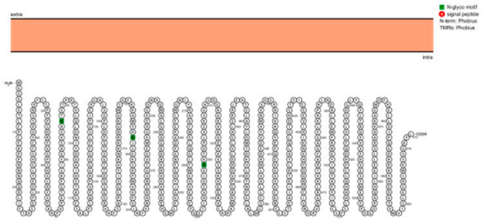
PtrPP2C24	Potri.003G159600v3.0	7	No	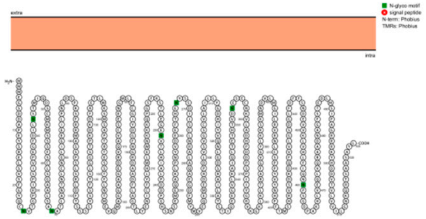

PtrPP2C25	Potri.003G183800v3.0	4	No	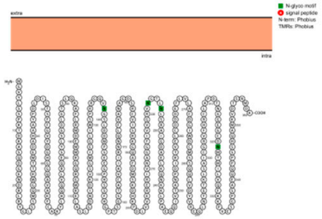
PtrPP2C26	Potri.004G066200v3.0	3	No	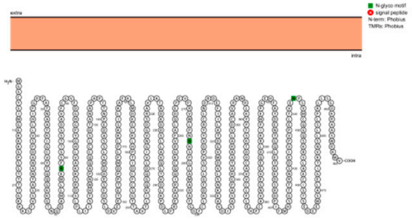
PtrPP2C27	Potri.004G177100v3.0	3	No	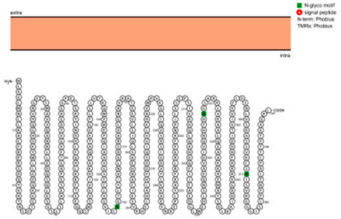
PtrPP2C28	Potri.005G021200v3.0	0	No	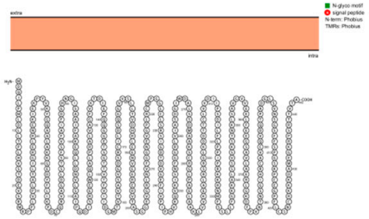

PtrPP2C29	Potri.005G021900v3.0	0	No	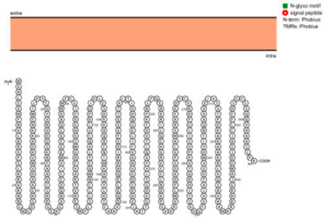
PtrPP2C30	Potri.005G102500v3.0	4	No	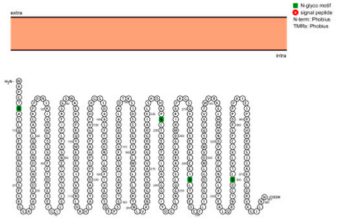
PtrPP2C31	Potri.005G108500v3.0	1	No	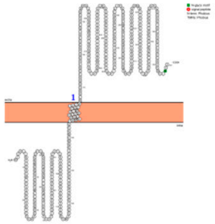
PtrPP2C32	Potri.005G125700v3.0	2	No	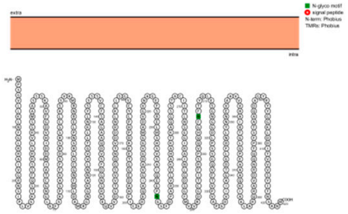

PtrPP2C33	Potri.005G160600v3.0	1	No	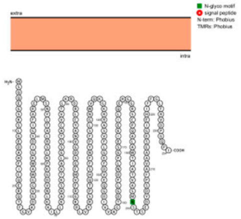
PtrPP2C34	Potri.005G164600v3.0	3	No	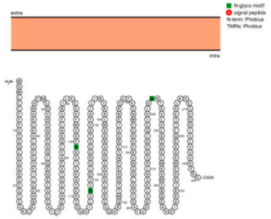
PtrPP2C35	Potri.005G186000v3.0	3	No	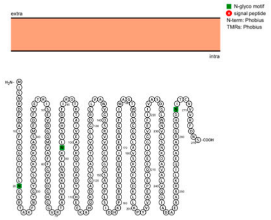
PtrPP2C36	Potri.005G214500v3.0	6	No	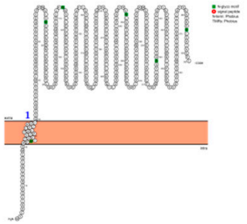

PtrPP2C37	Potri.005G214700v3.0	1	Yes	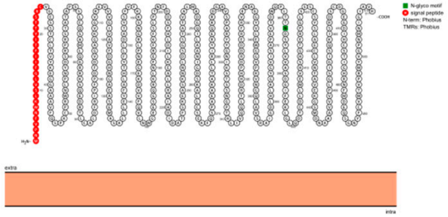
PtrPP2C38	Potri.005G253700v3.0	6	No	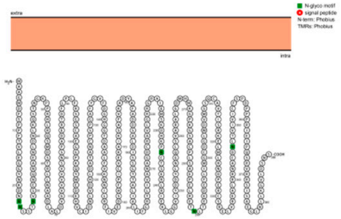
PtrPP2C39	Potri.006G059600v3.0	2	No	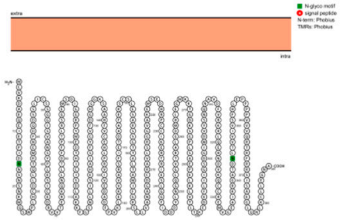
PtrPP2C40	Potri.006G081400v3.0	0	No	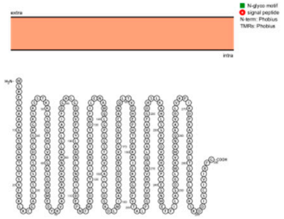

PtrPP2C41	Potri.006G085000v3.0	5	No	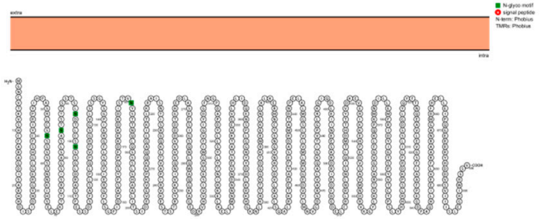
PtrPP2C42	Potri.006G105000v3.0	7	No	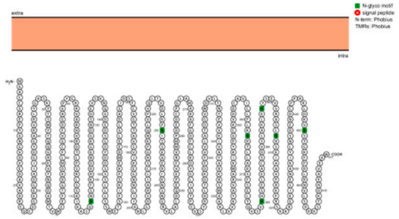
PtrPP2C43	Potri.006G134100v3.0	0	No	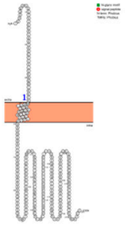
PtrPP2C44	Potri.006G164600v3.0	3	No	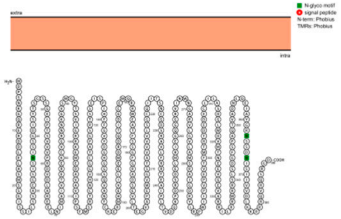

PtrPP2C45	Potri.006G192600v3.0	2	No	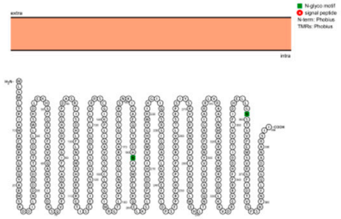
PtrPP2C46	Potri.006G224600v3.0	1	No	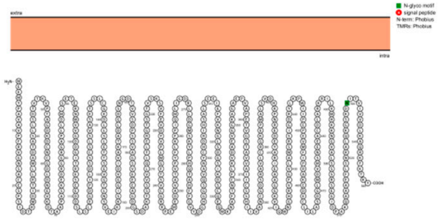
PtrPP2C47	Potri.006G232700v3.0	0	No	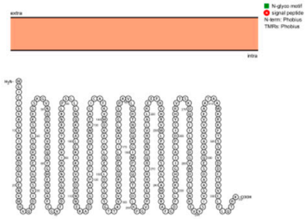
PtrPP2C48	Potri.006G248400v3.0	2	No	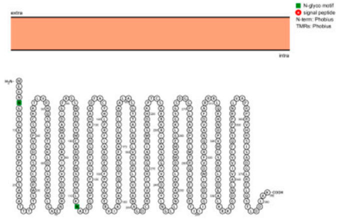

PtrPP2C49	Potri.006G265100v3.0	4	No	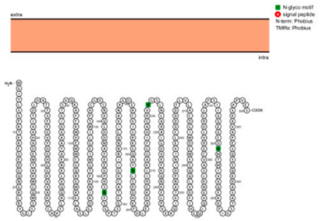
PtrPP2C50	Potri.006G267600v3.0	2	No	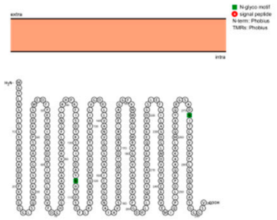
PtrPP2C51	Potri.007G028900v3.0	4	No	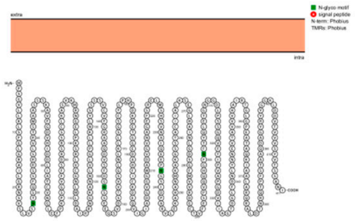
PtrPP2C52	Potri.007G051900v3.0	1	No	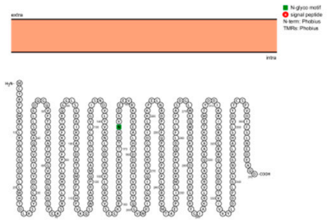

PtrPP2C53	Potri.007G058700v3.0	4	No	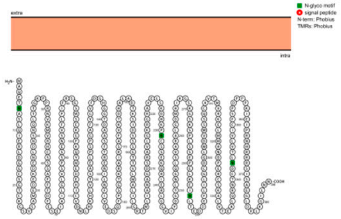
PtrPP2C54	Potri.007G061100v3.0	1	No	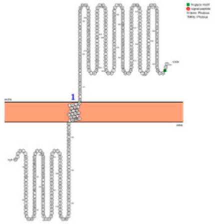
PtrPP2C55	Potri.008G046900v3.0	2	No	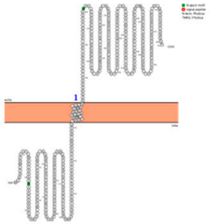
PtrPP2C56	Potri.008G059200v3.0	5	No	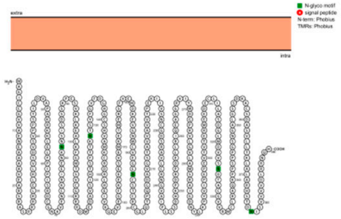

PtrPP2C57	Potri.008G070400v3.0	1	No	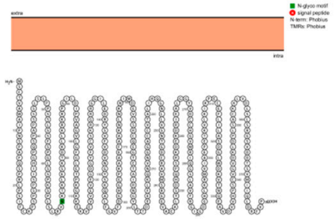
PtrPP2C58	Potri.008G100700v3.0	1	No	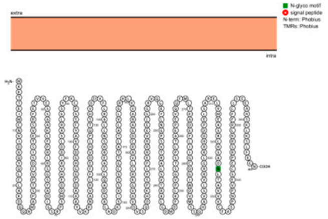
PtrPP2C59	Potri.008G104300v3.0	2	No	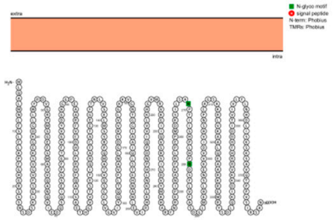
PtrPP2C60	Potri.008G123600v3.0	1	No	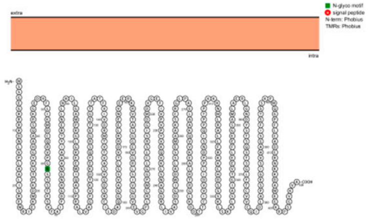

PtrPP2C61	Potri.008G149700v3.0	5	Yes	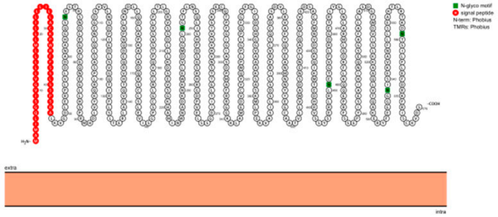
PtrPP2C62	Potri.008G168400v3.0	2	No	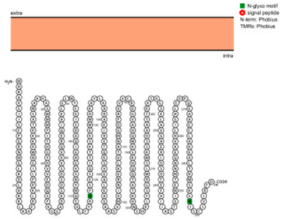
PtrPP2C63	Potri.008G198700v3.0	1	No	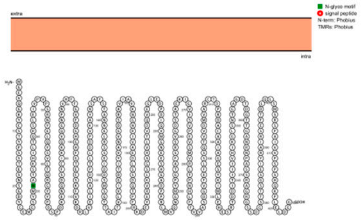
PtrPP2C64	Potri.008G207700v3.0	6	No	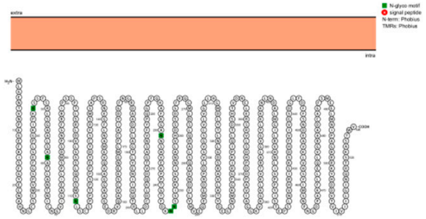

PtrPP2C65	Potri.008G209000v3.0	3	No	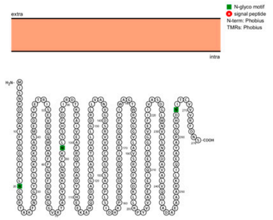
PtrPP2C66	Potri.009G021300v3.0	2	No	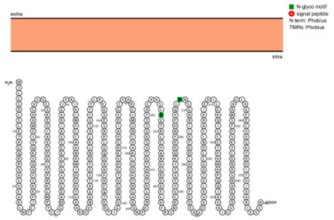
PtrPP2C67	Potri.009G030600v3.0	3	No	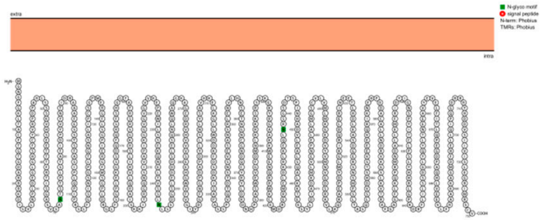
PtrPP2C68	Potri.009G037300v3.0	4	No	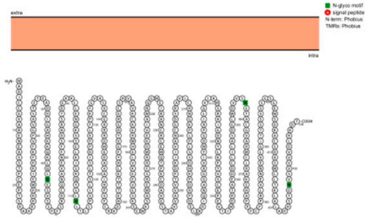

PtrPP2C69	Potri.009G073000v3.0	1	No	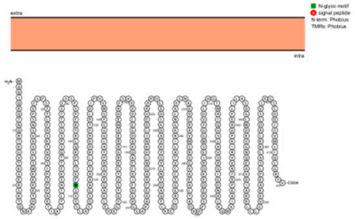
PtrPP2C70	Potri.009G091600v3.0	1	No	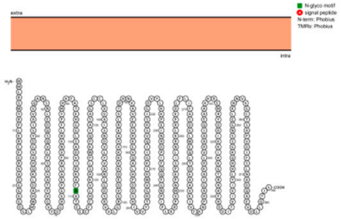
PtrPP2C71	Potri.009G137400v3.0	4	No	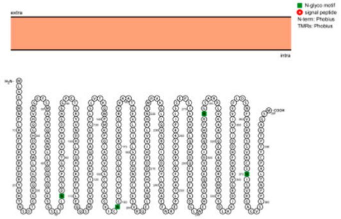
PtrPP2C72	Potri.010G006100v3.0	1	No	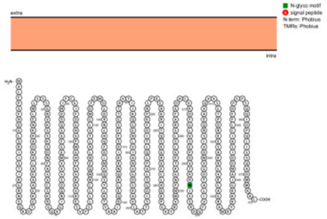

PtrPP2C73	Potri.010G006200v3.0	2	No	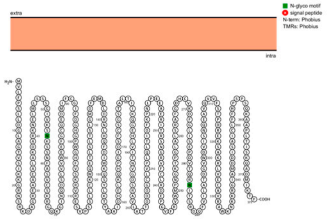
PtrPP2C74	Potri.010G009100v3.0	1	No	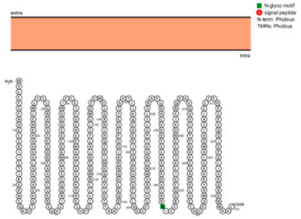
PtrPP2C75	Potri.010G009200v3.0	2	No	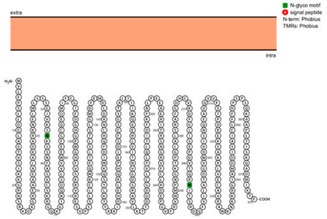
PtrPP2C76	Potri.010G024800v3.0	5	No	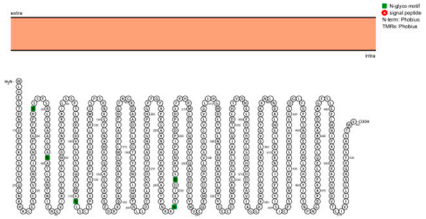

PtrPP2C77	Potri.010G028300v3.0	5	No	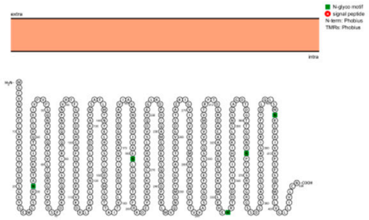
PtrPP2C78	Potri.010G047600v3.0	3	Yes	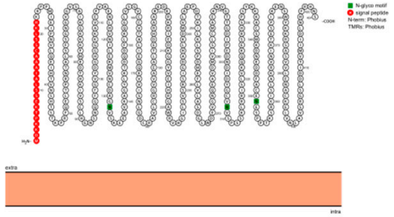
PtrPP2C79	Potri.010G070100v3.0	1	No	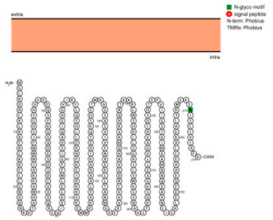
PtrPP2C80	Potri.010G091500v3.0	3	Yes	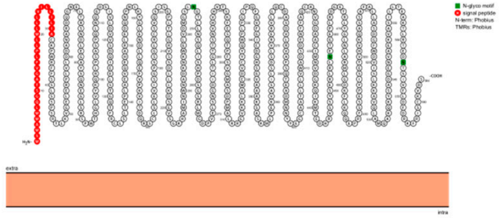

PtrPP2C81	Potri.010G121600v3.0	1	No	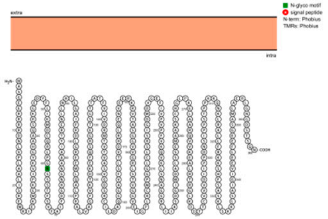
PtrPP2C82	Potri.010G146700v3.0	3	No	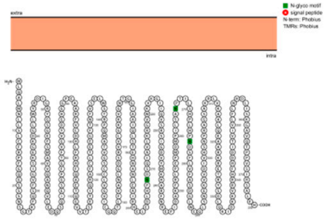
PtrPP2C83	Potri.010G151500v3.0	4	No	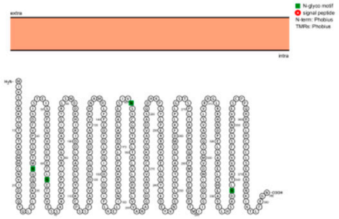
PtrPP2C84	Potri.010G187000v3.0	1	No	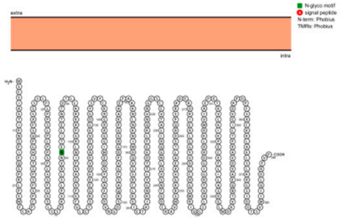

PtrPP2C85	Potri.010G199600v3.0	6	No	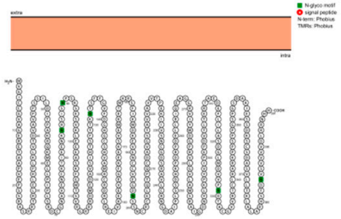
PtrPP2C86	Potri.010G214700v3.0	2	No	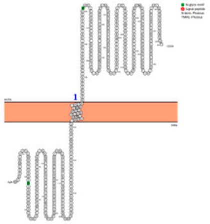
PtrPP2C87	Potri.011G013000v3.0	3	No	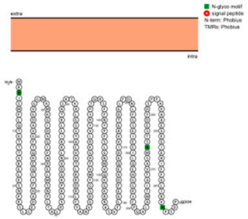
PtrPP2C88	Potri.011G102200v3.0	1	No	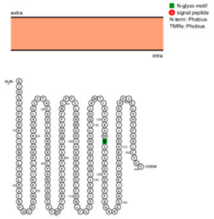

PtrPP2C89	Potri.011G116700v3.0	1	No	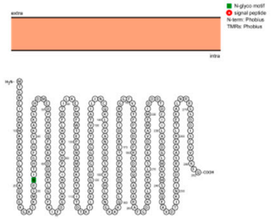
PtrPP2C90	Potri.012G002100v3.0	4	No	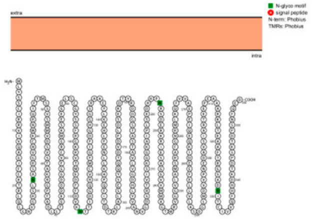
PtrPP2C91	Potri.012G002700v3.0	2	No	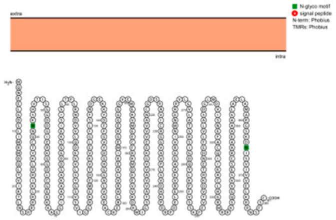
PtrPP2C92	Potri.012G131800v3.0	1	No	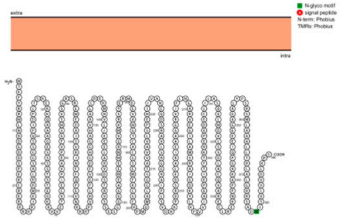

PtrPP2C93	Potri.013G011600v3.0	1	No	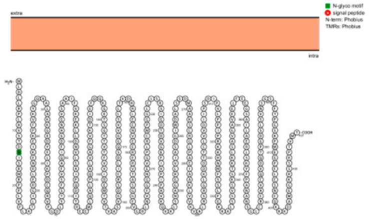
PtrPP2C94	Potri.013G012200v3.0	0	No	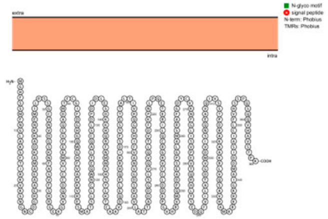
PtrPP2C95	Potri.013G085500v3.0	5	No	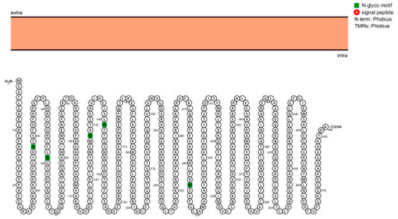
PtrPP2C96	Potri.013G099400v3.0	2	Yes	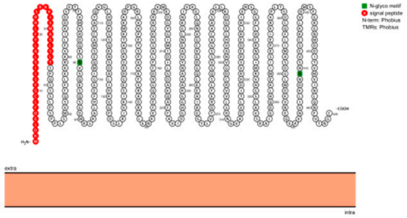

PtrPP2C97	Potri.013G144100v3.0	4	No	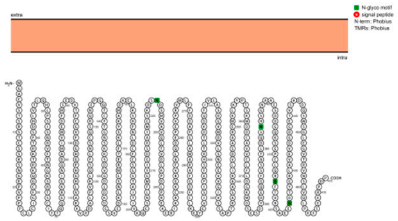
PtrPP2C98	Potri.014G031200v3.0	1	No	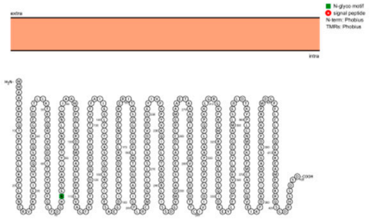
PtrPP2C99	Potri.014G031500v3.0	1	No	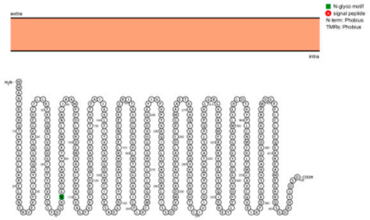
PtrPP2C100	Potri.014G042800v3.0	4	No	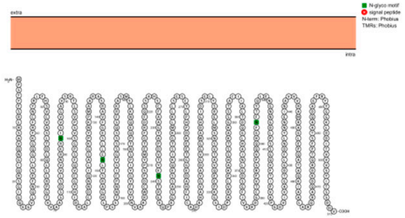

PtrPP2C101	Potri.014G110500v3.0	9	No	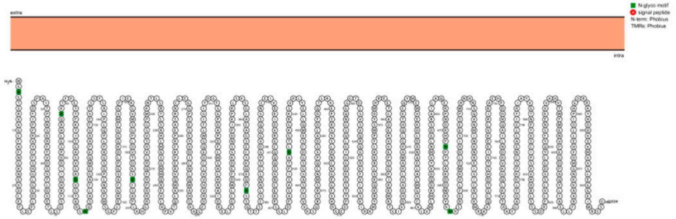
PtrPP2C102	Potri.014G115500v3.0	2	No	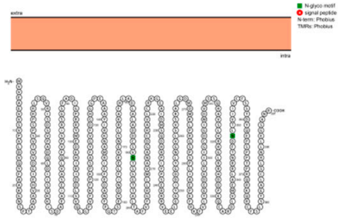
PtrPP2C103	Potri.015G010600v3.0	1	No	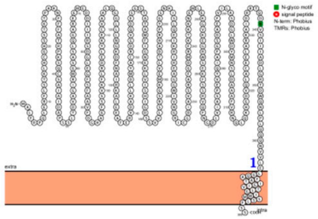
PtrPP2C104	Potri.015G018800v3.0	1	No	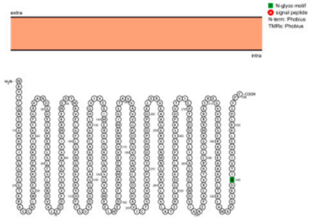

PtrPP2C105	Potri.015G019200v3.0	3	No	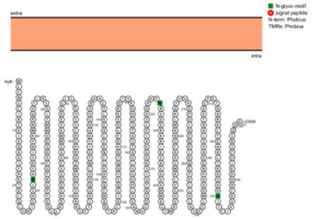
PtrPP2C106	Potri.015G043000v3.0	2	No	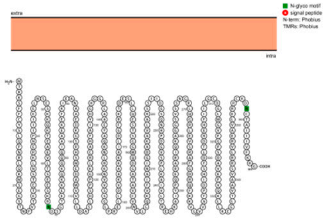
PtrPP2C107	Potri.015G133900v3.0	1	No	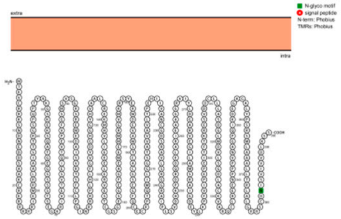
PtrPP2C108	Potri.016G045600v3.0	3	No	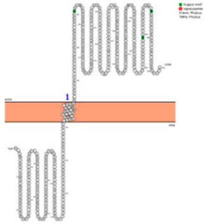

PtrPP2C109	Potri.016G082800v3.0	1	No	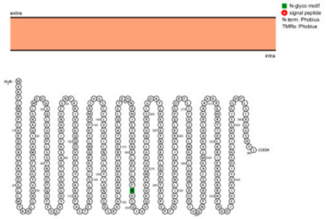
PtrPP2C110	Potri.016G127900v3.0	5	No	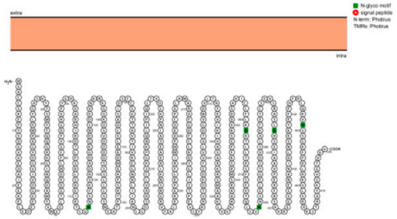
PtrPP2C111	Potri.017G013300v3.0	1	No	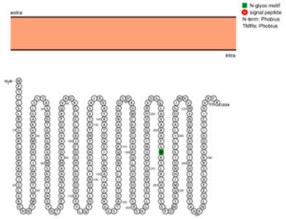
PtrPP2C112	Potri.017G023900v3.0	1	No	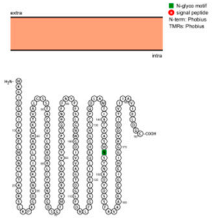

PtrPP2C113	Potri.018G013900v3.0	2	No	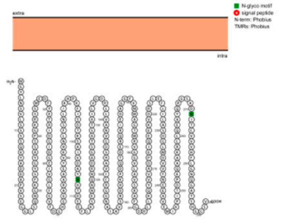
PtrPP2C114	Potri.018G017800v3.0	2	No	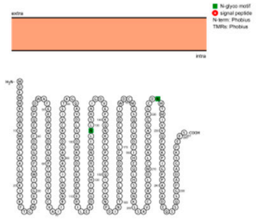
PtrPP2C115	Potri.018G033000v3.0	3	No	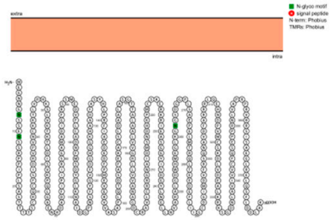
PtrPP2C116	Potri.018G059800v3.0	1	No	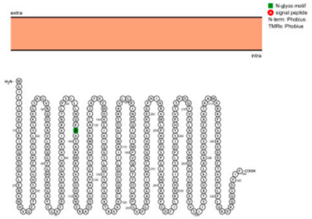

PtrPP2C117	Potri.018G060300v3.0	1	No	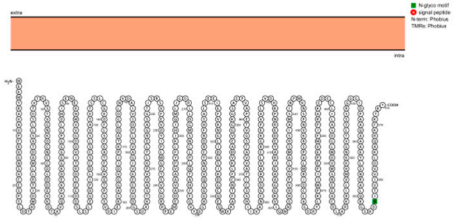
PtrPP2C118	Potri.018G115100v3.0	1	No	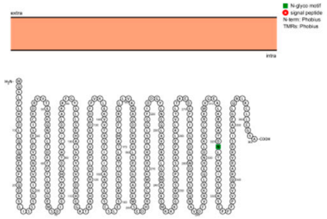
PtrPP2C119	Potri.018G150800v3.0	1	No	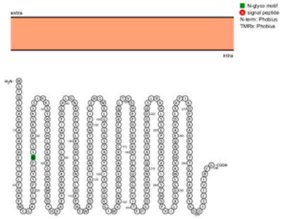
PtrPP2C120	Potri.019G054200v3.0	4	No	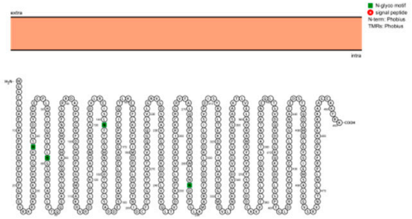

PtrPP2C121	Potri.019G071600v3.0	1	No	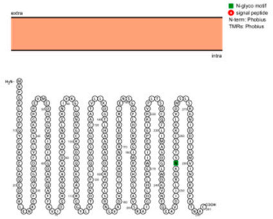
PtrPP2C122	Potri.019G103100v3.0	4	No	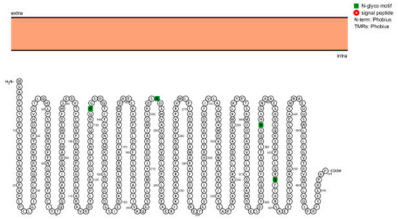
PtrPP2C123	Potri.T137100v3.0	1	No	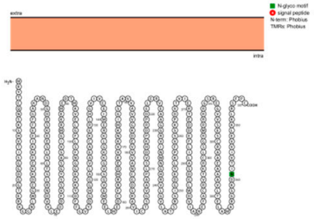
PtrPP2C124	Potri.T063000v3.0	3	No	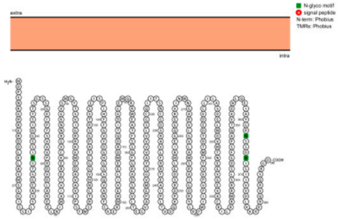
